# High-Throughput Protein Expression Using a Combination of Ligation-Independent Cloning (LIC) and Infrared Fluorescent Protein (IFP) Detection

**DOI:** 10.1371/journal.pone.0018900

**Published:** 2011-04-26

**Authors:** Hakan Dortay, Usha Madhuri Akula, Christin Westphal, Marie Sittig, Bernd Mueller-Roeber

**Affiliations:** 1 Institute of Biochemistry and Biology, University of Potsdam, Potsdam-Golm, Germany; 2 Max-Planck Institute of Molecular Plant Physiology, Potsdam-Golm, Germany; University of Crete, Greece

## Abstract

Protein expression in heterologous hosts for functional studies is a cumbersome effort. Here, we report a superior platform for parallel protein expression *in vivo* and *in vitro*. The platform combines highly efficient ligation-independent cloning (LIC) with instantaneous detection of expressed proteins through N- or C-terminal fusions to infrared fluorescent protein (IFP). For each open reading frame, only two PCR fragments are generated (with three PCR primers) and inserted by LIC into ten expression vectors suitable for protein expression in microbial hosts, including *Escherichia coli*, *Kluyveromyces lactis*, *Pichia pastoris*, the protozoon *Leishmania tarentolae*, and an *in vitro* transcription/translation system. Accumulation of IFP-fusion proteins is detected by infrared imaging of living cells or crude protein extracts directly after SDS-PAGE without additional processing. We successfully employed the LIC-IFP platform for *in vivo* and *in vitro* expression of ten plant and fungal proteins, including transcription factors and enzymes. Using the IFP reporter, we additionally established facile methods for the visualisation of protein-protein interactions and the detection of DNA-transcription factor interactions in microtiter and gel-free format. We conclude that IFP represents an excellent reporter for high-throughput protein expression and analysis, which can be easily extended to numerous other expression hosts using the setup reported here.

## Introduction

Genome sequencing has led to the discovery of myriads of new open reading frames from microbial, plant and animal systems whose cellular and biochemical functions are often unknown. Analysis of such proteins generally involves their expression in heterologous hosts, followed by their purification and biochemical characterization. However, expression of proteins in alien hosts is often a difficult and time-consuming task, requiring laborious screens to identify the optimal expression organism (or strain) and experimental setup. The situation is further complicated by the fact that plasmids needed for the transformation of the host strains are in most cases divergent with respect to their multi-cloning sites, requesting individual and often complicated (multi-step) cloning procedures for the insertion of a given open reading frame into different expression vectors. The establishment of rapid cloning, expression and protein detection procedures has therefore become a major field of interest for the design of high-throughput methods for parallel expression of proteins in multiple expression systems.

To serve rapid cloning, several technologies were established in recent years including e.g. the commercial Gateway (Invitrogen) [1], [2] and Creator (Clontech) [3] recombination systems and the proprietary In-Fusion assembly technology (Clontech) likely based on the 3′->5′ exonuclease activity of poxvirus DNA polymerase generating complementary 15-bp overhangs between target and destination DNA molecules [4]. Additionally, novel restriction enzyme/DNA ligase-mediated vector construction methods were established including BioBrick assembly (http://biobricks.org/) and Golden Gate cloning [5], [6].

Ligation-independent cloning (LIC), sometimes also referred to as ligase-independent cloning, is a simple, rapid and relatively cheap method for the generation of expression constructs. It uses the 3′->5′ exonuclease activity of T4 DNA polymerase to create specific single-stranded, 5′-extending tails of ∼10–18 nucleotides in DNA fragments (e.g. PCR amplicons) and complementary single-stranded overhangs in the target vector. Fragment and vector are mixed and annealed to each other in the absence of ligases. Circularization of the vector can only occur after insertion of the DNA fragments through their cohesive ends. The circular vector-fragment-annealed DNA is then transformed into *Escherichia coli*, where the newly established plasmids will replicate [7], [8]. LIC-compatible vectors contain specifically designed segments (LIC sites) into which the incoming fragments are cloned.

LIC-compatible vectors have recently been described for various experimental frameworks, including the high-throughput production of recombinant human proteins for crystal structure determination in bacteria [9], the generation of intron-containing hairpin RNA constructs for RNAi in plants [10], and the rapid construction of vectors for targeted mutagenesis in mycobacteria [11].

Next to cloning efficiency, the detection of proteins expressed in heterologous hosts represents a further experimental challenge. Although high-throughput protein expression has been described for e.g. *E. coli* [12], [13], [14], [15], insect cells [16], mammalian cells [17], [18] and for *in vitro* systems [19], rapid and cheap detection of recombinantly expressed proteins is still a time-consuming factor and remains a major bottleneck for multi-parallel expression of large numbers of different proteins.

Recently, infrared fluorescent protein (IFP) has been engineered as a new reporter protein, derived from a bacterial (*Deinococcus radiodurans*) phytochrome [20]. IFP covalently incorporates biliverdin, a natural product of heme catabolism involved in aerobic respiration, and becomes infrared fluorescent with excitation and emission maxima at 684 nm and 708 nm, respectively. Successful expression of IFP has been reported for *E. coli*, human embryonic kidney cells (HEK293A) and mice [20]. Recently, we demonstrated that IFP also functions as an excellent reporter for protein expression in *Leishmania tarentolae* [21], a unicellular eukaryotic protozoan for recombinant protein production [22].

**Figure 1:**
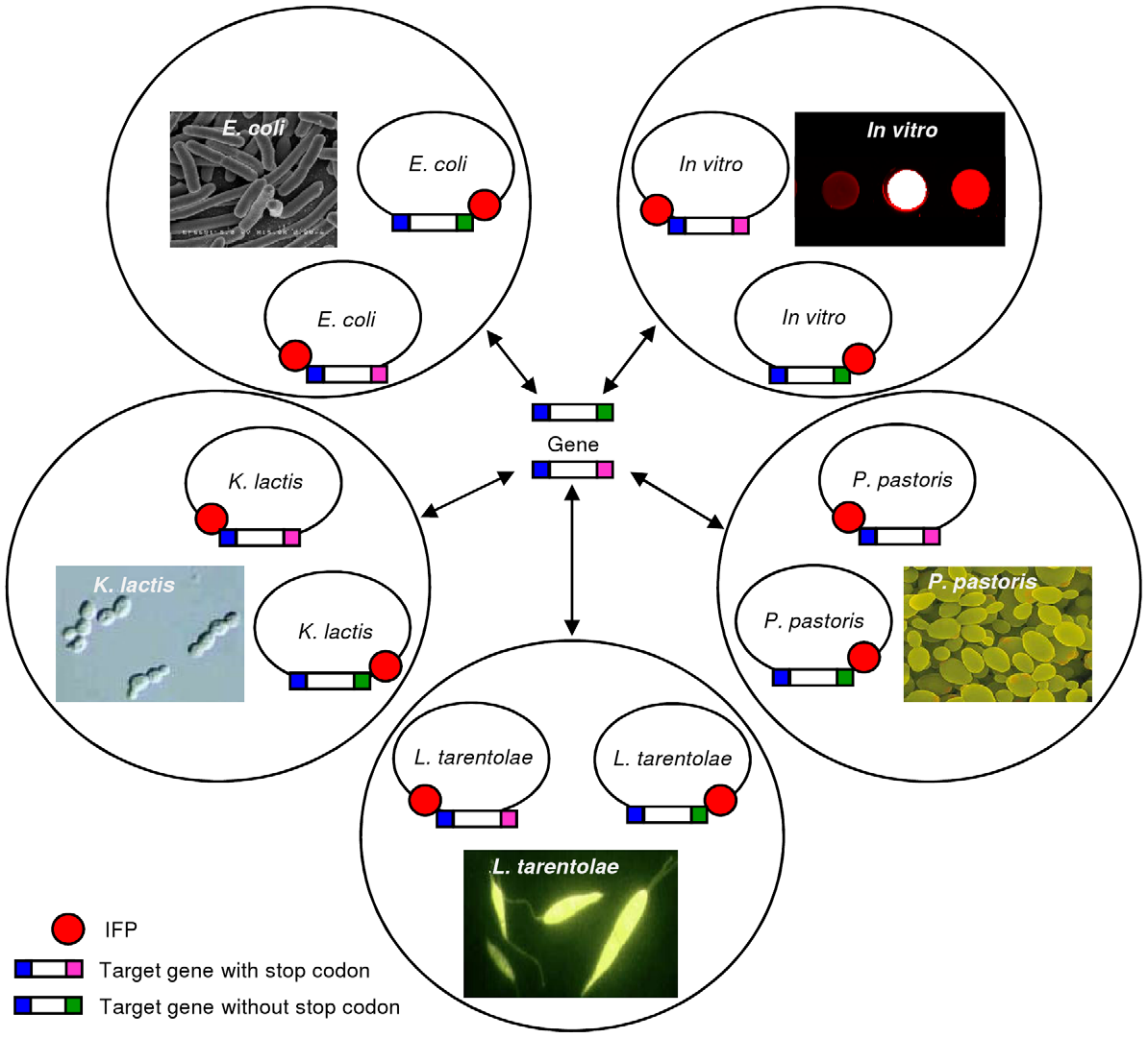
Rapid and parallel cloning using LIC-compatible expression vectors. Only two PCR fragments, one with and one without stop codon, are needed per target open reading frame for rapid and parallel insertion into ten LIC-compatible vectors. The vectors allow facile protein expression in four different hosts, i.e. *E. coli*, *K. lactis*, *P. pastoris* and *L. tarentolae*, and by *in vitro* transcription/translation. Vectors were constructed to support production of N- and C-terminal fusions to the IFP- and 6xHis-tags. The IFP moiety enables detection of IFP fusion proteins by easy-to-handle in-cell and in-gel infrared imaging, and the 6xHis-tag allows immunological detection of fusion proteins and affinity purification. A TEV protease cleavage site (not indicated) allows removal of the IFP- and 6xHis-tags. Photographs provided by: Wikipedia (*E. coli*); Linda Silveira, University of Redlands, California, USA (*K. lactis*); Dennis Kunkel, Dennis Kunkel Microscopy, Inc., Hawaii, USA (*P. pastoris*); Jena Bioscience, Jena, Germany (*L. tarentolae*).

Generally, vectors for heterologous protein expression are only partly standardized, which complicates strategies for expression of proteins in multiple hosts. Here, we decided to combine the benefits of LIC (efficient and rapid cloning) and IFP (suitability for in-cell and in-gel detection) for protein expression in multiple expression systems in high-throughput. We chose to generate LIC-compatible vectors for protein expression in *Escherichia coli* and *Pichia pastoris* [23]. According to recent data 80% of all recombinant proteins are currently expressed in these two organisms. However, as these expression systems are often inadequate for expression of eukaryotic proteins, the use of alternative and less frequently used systems has been recommended [24]. We therefore included the yeast *Kluyveromyces lactis* [25] and the protozoan *Leishmania tarentolae* [22] as two additional, eukaryotic expression hosts in our setup. Finally, the LIC-compatible cloning system was also established for *in vitro* protein expression (**Fig. 1**).

To demonstrate the capacity of our platform we generated ten LIC-compatible vectors for oriented insertion of open reading frames and then built 54 constructs for the expression of eight different plant and two fungal proteins, including transcription factors and enzymes, in the five production systems. All vectors support the expression of proteins with either N- or C-terminal fusions to the IFP reporter and a 6xHis-tag enabling rapid identification of well-expressing host strains or *in vitro* expression conditions by in-cell, in-gel and immunological detection as well as protein purification by affinity chromatography. Additionally, the marker proteins can be cleaved off by treatment with Tobacco Etch Virus (TEV) protease recognizing a cognate TEV cleavage site [26] included in all proteins.

With the vectors generated here we observed 100% cloning efficiency in almost all experiments, i.e. virtually all LIC-inserted PCR fragments were present in correct orientation after restriction analysis and were free of sequencing errors and out-of-frame fusions after sequencing. Additionally, IFP-labelled fusion proteins were detected in all cases, eight *in vitro*, eleven in *E. coli*, five in *K. lactis*, four in *P. pastoris* and seven in *L. tarentolae*. Four IFP fusion proteins expressed in *E. coli* were used for functionality analysis, resulting in successful purification by 6xHis affinity chromatography and time-dependent TEV protease cleavage of the 6xHis and IFP reporter proteins. Our platform, which requires minimal effort for designing appropriate cloning strategies, allows for simple screening of optimal expression systems and provides a fertile tool for proteomics research. As examples we demonstrate that IFP fusion proteins can be employed for *in vitro* protein-protein interaction studies as well as for the analysis of DNA-transcription factor interactions, making IFP fusions amenable to high-throughput screening processes. IFP fusion proteins are conveniently detected by infrared imaging in microtiter plates, or after SDS-polyacrylamide gel electrophoresis in cast protein gels. After pull-down, IFP fusion proteins can thus be directly visualized by infrared imaging making additional experimental steps such as western blotting or autoradiography of radioactively labelled proteins frequently used in such studies [27], [28] obsolete. Finally, we demonstrated enzymatic activity of two selected IFP fusion proteins. Our IFP fusion protein tool box offers an easy-to-handle platform for protein expression and facilitates the analysis of protein-protein and protein-DNA interactions.

## Materials and Methods

### Chemicals

Biliverdin hydrochloride was purchased from Frontier Scientific (Carnforth, Lancashire, UK). Hemin was ordered from Sigma-Aldrich (Deisenhofen, Germany).

### Constructs

#### General

**Figure 2:**
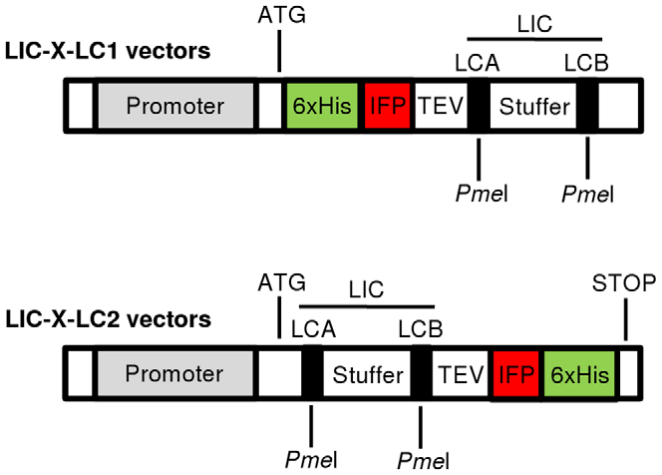
LIC-compatible expression vectors. The pool of LIC-compatible vectors comprises vectors for expression of IFP fusion proteins in multiple expression systems, i.e. *in vitro* (LIC-pIVEX-LC1/-LC2) and *in vivo* in *E. coli* (LIC-pDEST-LC1/-LC2), *K. lactis* (LIC-pKLAC-LC1/-LC2), *P. pastoris* (LIC-pPICZ-LC1/-LC2) and *L. tarentolae* (LIC-pLEXSY-LC1/-LC2). The pool includes LC1 and LC2 vectors encoding 6xHis-IFP-TEV-ProteinX and ProteinX-TEV-IFP-6xHis fusions, respectively, after ligation-independent cloning of ProteinX-encoding open reading frames into the LCA and LCB sites. The maker proteins IFP and 6xHis can be cleaved off at the TEV protease cleavage site next to a PmeI site used for LIC. LCA and LCB sites are introduced into target open reading frames by PCR. The LIC fragment was designed on the basis of a 670-bp stuffer fragment, flanked by the LIC annealing sites LCA and LCB, respectively, both of which encompass a PmeI restriction site. The stop codon of LC2 vectors is provided by the vector whilst for LC1 vectors it has to be added by the reverse primer during PCR amplification of the target open reading frame.

IFP-LIC compatible *in vitro*, *E. coli*, *K. lactis*, *P. pastoris* and *L. tarentolae* protein expression vectors were generated by using the commercial vectors pIVEX2.4d/2.3d (Roche, Mannheim, Germany), pDEST15 (Invitrogen, Karlsruhe, Germany), pKLAC1 (New England Biolabs, Frankfurt am Main, Germany), pPICZ-αA (Invitrogen), and pLEXSY-sat2 (Jena Bioscience, Jena, Germany). The IFP-LIC vectors were generally denoted as LIC-X-LC1 or LIC-X-LC2, where ‘X’ refers to the original vectors (pIVEX, pDEST, pKLAC, pPICZ and pLEXSY), that were used to construct the LIC-compatible vectors (**Fig. 2**). ‘LC1’ indicates positioning of the LIC site downstream of the 6xHis-IFP-TEV fusion segment (downstream of the C-terminus of the TEV protease cleavage site). ‘LC2’ indicates the LIC site to be located upstream of the TEV-IFP-6xHis fusion segment (upstream of the N-terminus of the TEV cleavage site). The LIC fragment is identical in all vectors and was designed on the basis of a stuffer fragment derived from the *L. tarentolae* expression vector pLEXSY-sat2 (Jena Bioscience) to which we added by PCR the LIC annealing sites LCA and LCB at both ends. Both LIC sites include a PmeI restriction site (**Fig. 2**). For PCR amplification of the IFP open reading frame the pENTR1A-IFP1.4&GFP vector [20] was used as template. IFP-LIC compatible vectors were generated as described below (for primer sequences see **Table S1**). Vector sequences were deposited in GenBank under the following accession numbers: LIC-pIVEX-LC1, JF327844; LIC-pIVEX-LC2, JF327845; LIC-pDEST-LC1, JF327846; LIC-pDEST-LC2, JF327847; LIC-pKLAC-LC1, JF327848; LIC-pKLAC-LC2, JF327849; LIC-pPICZ-LC1, JF327850; LIC-pPICZ-LC2, JF327851; LIC-pLEXSY-LC1, JF327852; LIC-pLEXSY-LC2, JF327853.

#### LIC-pIVEX-LC1 – *in vitro* expression vector

The IFP open reading frame was amplified by PCR using primers P294 and P295. The LIC sequence was amplified using primers P296 and P297. Subsequently, the PCR products were used for fusion-PCR with primers P294 and P297. The final PCR product, called IFP-TEV-LIC, was cloned by restriction and ligation into the NotI and BamHI sites of the pIVEX2.4d vector, resulting in the *in vitro* expression vector LIC-pIVEX-LC1 encoding for N-terminal 6xHis-FactorXa-IFP-TEV fusion proteins if LIC is performed.

#### LIC-pIVEX-LC2 - *in vitro* expression vector

The LIC fragment was amplified by PCR using primers P298 and P299. The IFP open reading frame was amplified using primers P300 and P283. Both PCR products were used in a fusion-PCR with primers P298 and P283. The fusion-PCR product, called LIC-TEV-IFP, was cloned by restriction and ligation into the NcoI and SmaI sites of the pIVEX2.3d vector, resulting in the *in vitro* expression vector LIC-pIVEX-LC2 encoding for C-terminal TEV-IFP-6xHis fusion proteins if LIC is performed.

#### LIC-pDEST-LC1 – *E. coli* expression vector

This vector was generated by amplification of 6xHis-IFP coding region in a two-step PCR. For the first reaction, PCR primers P301a and P295 were used. The resulting PCR product was used as template for the second PCR with primers P301b and P295, yielding fragment 6xHis-IFP. The LIC sequence, which was later used for the fusion-PCR with the 6xHis-IFP PCR product, was amplified with primers P296 and P297. Fusion-PCR with the PCR products 6xHis-IFP and LIC was done using primers P301b and P297, resulting in the PCR product 6xHis-IFP-TEV-LIC. This product was cloned by restriction and ligation into the NdeI and BamHI sites of the vector pDEST15 (Invitrogen). The resulting *E. coli* expression vector was named LIC-pDEST-LC1; it encodes for N-terminal 6xHis-IFP-TEV fusion proteins if LIC is performed.

#### LIC-pDEST-LC2 – *E. coli* expression vector

This vector was generated by using the pHEST-IFP vector encoding for the IFP-6xHis fusion protein. The pHEST-IFP vector was generated by cloning of IFP-6xHis encoding sequence, with a KpnI restriction site between the IFP and the 6xHis moieties, into the NdeI and BamHI sites of the pDEST15 vector. For the amplification of the LIC fragment, PCR primers P302 and P299 were used. IFP was amplified using primers P300 and P303. The resulting PCR products were used for fusion-PCR with primers P302 and P303 to generate the LIC-TEV-IFP-6xHis cassette for cloning by restriction and ligation into the NdeI and KpnI sites of the vector pHEST-IFP. The resulting *E. coli* expression vector was called LIC-pDEST-LC2 encoding for C-terminal TEV-IFP-6xHis fusion proteins if LIC is performed.

#### LIC-pKLAC-LC1 - *K. lactis* expression vector

This vector was generated by amplification of 6xHis-IFP in a two-step PCR reaction. Primers P301a and P295 were used for the first reaction. The resulting PCR product was then employed as template for the second PCR with primers P312 and P295, resulting in fragment 6xHis-IFP. The LIC fragment, later used for the fusion-PCR with the 6xHis-IFP PCR product, was amplified with primers P296 and P305. Fusion-PCR with the PCR products 6xHis-IFP and LIC was done using primers P312 and P305. The resulting PCR-product was named 6xHis-IFP-TEV-LIC. This product was cloned by restriction and ligation into the HindIII and NotI sites of the vector pKLAC1 (NEB). The resulting *K. lactis* expression vector was named LIC-pKLAC-LC1 encoding for N-terminal 6xHis-IFP-TEV fusion proteins if LIC is performed.

#### LIC-pKLAC-LC2 - *K. lactis* expression vector

The LIC fragment was PCR amplified with primers P313 and P299. The IFP-6xHis sequence was amplified in a two-step PCR reaction. For the first reaction, PCR primers P300 and P314a were used. The resulting PCR product was used as template for the second PCR with primers P300 and P314b, resulting in fragment IFP-6xHis. Fusion-PCR with the PCR products LIC and IFP-6xHis was done using primers P313 and P314b, resulting in the PCR-product LIC-TEV-IFP-6xHis. This product was cloned by restriction and ligation into the HindIII and NotI sites of the vector pKLAC1. The resulting *K. lactis* expression vector was named LIC-pKLAC-LC2 encoding for C-terminal TEV-IFP-6xHis fusion proteins if LIC is performed.

#### LIC-pPICZ-LC1 - *P. pastoris* expression vector

Before generating the LIC-IFP compatible *P. pastoris* expression vectors (LIC-pPICZ-LC1 and LIC-pPICZ-LC2) a PmeI restriction site located within the 5′ *AOX1* promoter (at position 410–418 bp) of the pPICZ-αA vector was eliminated by changing the PmeI restriction site GTTTAAAC to GTTTAAAG. To this end, the vector was digested with PmeI and BstXI resulting in two fragments (3300 bp and 293 bp). The short fragment was used as template for PCR amplification with primers F-NoPmeI, (5′-AAAGGCTGTCTTGGAACC-3′) and R-NoPmeI (5′-ATAAGAATCCAGAATCTTGGAA-GCATAC-3′) to produce a DNA fragment lacking the PmeI restriction site. This PCR product was digested with BstXI and ligated back into the large 3300-bp fragment.

The resulting pPICZ-αA vector lacking the PmeI restriction site (named pPICZ-αA-ΔPme) was used to generate the LIC-pPICZ-LC1 vector by PCR amplification of the 6xHis-IFP fragment in a two-step PCR. For the first reaction, PCR primers P301a and P295 were used. The resulting PCR product was used as template for the second PCR with primers P308 and P295, resulting in the fragment 6xHis-IFP. The LIC fragment was amplified with primers P269 and P309. Fusion-PCR with the PCR products 6xHis-IFP and LIC was done using primers P308 and P309, resulting in the PCR product 6xHis-IFP-TEV-LIC. This product, digested with AclI and SalI, was cloned by restriction and ligation into the BstBI and SalI sites of the vector pPICZ-αA-ΔPme. The resulting *P. pastoris* expression vector was named LIC-pPICZ-LC1 encoding for N-terminal 6xHis-IFP-TEV fusion proteins if LIC is performed.

#### LIC-pPICZ-LC2 - *P. pastoris* expression vector

The LIC fragment was amplified by PCR with the primers P310 and P299, and the IFP open reading frame with primers P300 and P311. Fusion-PCR with the PCR products LIC and IFP was done using primers P310 and P311. The resulting PCR product was named LIC-TEV-IFP and cloned after AclI and SalI digestion by restriction and ligation into the BstBI and SalI sites of the pPICZ-αA-ΔPme vector. The resulting *P. pastoris* expression vector was named LIC-pPICZ-LC2 encoding for C-terminal TEV-IFP-6xHis fusion proteins if LIC is performed.

#### LIC-pLEXSY-LC1 - *L. tarentolae* expression vector

The 6xHis-IFP fragment was amplified by PCR in a two-step PCR using primers P301a and P295 in the first reaction. The resulting PCR product was used as template for the second PCR with primers P304 and P295. The LIC fragment was amplified with primers P296 and P305. Both PCR products were used in a fusion-PCR with primers P304 and P305. The fusion-PCR product, called 6xHis-IFP-TEV-LIC, was cloned by restriction and ligation into the NcoI and NotI sites of the pLEXSY-sat2 vector, resulting in the *L. tarentolae* expression vector LIC-pLEXSY-LC1 encoding for N-terminal 6xHis-IFP-TEV fusion proteins if LIC is performed.

#### LIC-pLEXSY-LC2 - *L. tarentolae* expression vector

The LIC fragment was amplified by PCR with primers P298 and P299. The IFP fragment was amplified by PCR with primers P300 and P303. The resulting PCR products were used in a fusion-PCR with primers P298 and P303. The fusion-PCR product, called LIC-TEV-IFP, was cloned by restriction and ligation into the NcoI and KpnI sites of the pLEXSY-sat2 vector. The resulting *L. tarentolae* expression vector was called LIC-pLEXSY-LC2 encoding for C-terminal TEV-IFP-6xHis fusion proteins if LIC is performed.

### Ligation-independent cloning

#### Linearization of LIC expression vectors for LIC cloning

LIC expression vectors (10 µg) were cut with 10 U PmeI in a 20-µL reaction volume and purified from contaminating stuffer fragment and undigested vector by gel-extraction using the NucleoSpin Extract II kit (Macherey & Nagel, Düren, Germany). To generate 5′ LIC overhangs (15 and 16 nt, respectively) at both ends the purified vector backbone was treated for 30 min (22°C) with T4 DNA polymerase in the presence of dATP, using the following reaction setup: 0.2 pmol purified vector backbone, 2 µL 10× buffer 2 (NEB), 2 µL dATP (25 mM), 1 µL dithiothreitol (DTT, 100 mM), 2 µL 10× (10 mg/mL) bovine serum albumin (BSA; NEB), 10 U T4 DNA polymerase (NEB) in a volume of 20 µL (filled up with ddH_2_O). The reaction mix was heat inactivated for 20 min at 75°C, followed by purification using the NucleoSpin Extract II kit (Macherey & Nagel) and elution with 20 µL elution buffer included in the kit. Two-µL aliquots were stored at 20°C before LIC cloning was performed.

#### Primer design and preparation of PCR products for LIC cloning

For directional and in-frame cloning of PCR-amplified open reading frames into LIC-X-LC1 or LIC-X-LC2 vectors, sense primers starting with the following sequence were used: 5′-TGGGTTCTTCTGTTTCC(ATG)-3′ (the ATG nucleotides in brackets indicate the gene's start codon). Antisense primers for cloning into LIC-X-LC1 expression vectors must start with the sequence 5′-GGTTCTCGCCCTGTTTACC(CTATTA)-3′ (brackets indicate a TAATAG double stop codon). Antisense primers for cloning into LIC-X-LC2 expression vectors must start with the sequence 5′-GGTTCTCGCCCTGTTTACC-3′ lacking a stop codon. Complementary LIC overhangs within the sense and antisense primers are underlined (for full primer sequences see **Table S2**). The cDNAs encoding for the *Arabidopsis thaliana* proteins TPK1, SAM1, ACO1, ACS2, ANAC042, ANAC059, BGAL4 and BGAL10 were amplified by PCR, respectively, using cDNAs of TPK1 (AGI: At5g55630), SAM1 (At1g02500), ACO1 (At2g19590), ACS2 (At1g01480), ANAC042 (At2G43000), and ANAC059 (At3g29035) as templates. BGAL4 (At5g56870) and BGAL10 (At5g63810) encoding vectors (pda07078 and pda08126) were purchased from the RIKEN Bio Resource Centre (Japan). For TPK1, a partial cDNA encoding the N-terminal part of the channel protein (amino acids 1-79) was used [29]. Open reading frames of two cell wall degrading enzymes *endo-β-1,4-glucanase* (GenBank ID DQ490472) and *endo-β-1,4-xylanase* (DQ490490) were PCR amplified using genomic DNA from *Pichia pastoris* strains obtained from the Fungal Genetic Stock Centre (FGSC) [30]. PCR products were treated at 22°C for 30 min with T4 DNA polymerase in the presence of dTTP, using the following reaction setup: 0.2 pmol purified PCR product, 2 µL 10× buffer 2 (NEB), 2 µL dATP (25 mM), 1 µL DTT (100 mM), 2 µL 10× BSA (10 mg/mL; NEB), 1 U T4 DNA polymerase (NEB) in a volume of 20 µL (filled up with ddH_2_O). The reaction mix was heat inactivated for 20 min at 75°C, followed by purification using the NucleoSpin Extract II kit (Macherey &Nagel) and eluting with 20 µL elution buffer included in the kit. Two-µL aliquots were stored at −20°C before LIC cloning was performed.

#### LIC cloning of target genes

0.02 pmol (1 µL) and 0.04 pmol (2 µL) of pre-treated LIC vectors and PCR products (see above) were mixed and incubated for 1 h at 22°C. The reaction mix was supplemented with 1 µl EDTA (25 mM), followed by incubation for 10 min at 22°C and transformation of the whole reaction mix into *E. coli* for plasmid amplification.

### Gateway cloning of cDNAs encoding GRFs

The open reading frames of *GRF1* (AGI code: At4g09000), *GRF2* (At1g78300), *GRF3* (At5g38480), *GRF4* (At1g35160), *GRF5* (At5g16050) and *GRF6* (At5g10450) were amplified by PCR using respective cDNA clones as templates and recombined into entry vector pDONR201 using Gateway technology (Invitrogen). Primers containing the Gateway *att*B1 and *att*B2 sites are listed in **Table S3**. The identities of all cloned cDNAs were verified by sequencing. For protein expression and purification, *GRF1* – *GRF6* open reading frames were recombined *in vitro* from the entry vectors into the Gateway destination vector pDEST15 (Invitrogen) encoding an N-terminal GST-tag. The resulting expression vectors were named pDEST15-GRF1/2/3/4/5/6.

### Protein expression

#### Protein expression *in vitro*

For *in vitro* transcription/translation LIC-pIVEX-LC1/LC2 plasmid templates were purified using the NucleoSpin Plasmid miniprep kit (Macherey & Nagel). The RTS 100 *E. coli* HY kit (Roche) was used to set up 50-µL reactions in 1.5-mL plastic tubes. Reactions were incubated for 5 h at 30°C, followed by incubation for 30 min at 26°C in the presence of 25 µM biliverdin hydrochloride. Samples (10–20 µL) were separated by SDS-PAGE and analysed by in-gel detection or western blot and infrared analysis as described [21].

#### Protein expression in *E. coli* and TEV protease cleavage

For protein expression in *E. coli* LIC-pDEST-LC1/LC2 plasmid templates were transformed into different expression strains, i.e. BL21 (DE3) pLysS (Agilent Technologies, Waldbronn, Germany), BL21 (DE3) CodonPlus-RIL (Agilent Technologies), and Rosetta (DE3) pRARE (Merck, Darmstadt, Germany). Furthermore, plasmid pRARE was isolated from Rosetta (DE3) pRARE cells and used to transform *E. coli* BL21 Star (DE3) (Invitrogen) to generate the expression strain BL21 Star (DE3) pRARE. Expression of IFP fusion proteins was induced at 30°C in LB medium (2 mL in 24-deep-well plates) supplemented with 25 µM biliverdin hydrochloride by 1 mM isopropyl thio-β-D-galactoside (IPTG) for 4 h. Hundred µL of the induced cell cultures were then used for in-cell detection by infrared imaging. Cells from 1 mL of culture were harvested after 4 h of induction and lysed by sonication in 100 µL lysis buffer (20 mM sodium phosphate buffer, pH 7.3, 150 mM NaCl, 1 mM EDTA, 1 mM DTT, 1 mM phenylmethanesulfonyl fluoride (PMSF), 2 mM benzamidin, 10 µg mL^−1^ aprotonin, 10 µg mL^−1^ leupeptin). Cell extracts were ultracentrifuged and 20 µL of the pellet and supernatant were used for SDS-PAGE separation followed by in-gel detection or western blot and infrared analysis [21]. Protein expression for the purification of GST (empty pDEST15 vector) or GST-GRF fusion proteins (pDEST15-GRF1/2/3/4/5/6 vectors) was carried out in 100 mL culture volume in BL21 (DE3) pLysS (Agilent Technologies) cells (30°C, 1 mM IPTG, 4 h), followed by sonication of cells in 10 mL lysis buffer as described above. Supernatants of ultracentrifuged cell extracts were used for purification by GST affinity chromatography (see below). Expression for purification of 6xHis-IFP-TEV-SAM1 and -ACO1 as well as ANAC042- and ANAC059-TEV-IFP-6xHis fusion proteins was carried out in 100-mL culture volumes in BL21 (DE3) pLysS (6xHis-IFP-TEV-SAM1/–ACO1) or BL21 Star (DE3) pRARE (ANAC042- and ANAC059-TEV-IFP-6xHis) cells (30°C, 1 mM IPTG, 4 h) in the absence of biliverdin hydrochloride, followed by sonication of cells in 5 mL lysis buffer (see above) supplemented with 25 µM biliverdin hydrochloride and 0.1 mM EDTA. The low EDTA concentration (0.1 mM) was used instead of 1 mM to minimize damaging of the columns used for purification of 6xHis fusion proteins. Supernatant fractions of ultracentrifuged cell extracts were used for TEV cleavage experiments and protein purification. For TEV cleavage experiments 160 µL of the protein samples were supplemented with 0.4 mM EDTA and incubated with 10 U AcTEV-Protease (Invitrogen) at 26°C. After 1 h, 2 h and 4 h of incubation 40-µL aliquots were separated by SDS-PAGE and analyzed by in-gel detection followed by Coomassie staining.

#### Protein expression in *K. lactis*

LIC-pKLAC-LC1/LC2 plasmid templates were used for protein expression in *K. lactis* using the *K. lactis* Protein Expression Kit (NEB) as described in the manufacturer's instructions. After three, four and five days, respectively, of incubation 100 µL of galactose-induced IFP fusion protein-expressing cells were used for in-cell detection. To this end, cell cultures were supplemented with hemin (10 µg/mL) and incubated for further 2 h to trigger the formation of chromophore-attached IFP. The cell line producing the strongest infrared signal was scaled up in 100 mL culture volume for in-gel detection or western blot and infrared analysis. To this end, cells were harvested and lysed with 20 mL lysis buffer (see above, protein expression in *E. coli*) at 2,000 bar using an EmulsiFlex-C5 high-pressure homogenizer (Avestin Europe, Mannheim, Germany). Crude extracts were ultracentrifuged and 20 µL of the pellet and supernatant were used for SDS-PAGE separation followed by in-gel detection or western blot and infrared analysis [21].

#### Protein expression in *P. pastoris*

LIC-pPICZ-LC1/LC2 plasmid templates were used for protein expression in *P. pastoris* using the EasySelect *Pichia* Expression kit (Invitrogen), as described in the manufacturer's instructions. After three, four and five days, respectively, of incubation 100 µL of methanol-induced IFP fusion protein-expressing cells were used for in-cell detection. To this end, cell cultures were supplemented with hemin (10 µg/mL) and incubated for further 2 h to trigger the generation of infrared signal. The cell line producing the strongest infrared signal was scaled up in 100 mL culture volume for in-gel detection or western blot and infrared analysis after 24 h of induction with methanol. Cells were harvested and processed further as described above (protein expression in *K. lactis*).

#### Protein expression in *L. tarentolae*

LIC-pLEXSY-LC1/LC2 plasmid templates were used for protein expression in *L. tarentolae* using the LEXSYcon2 Expression Kit (Jena Bioscience) as described before [21]. One hundred µL of IFP fusion protein expressing cells were used for in-cell detection and 8 mL of harvested cells were lysed with 100 µL of lysis buffer (see above, protein expression in *E. coli*) and sonication after five days of incubation. Crude extracts were ultracentrifuged and 20 µL of the pellet and supernatant were used for SDS-PAGE separation followed by in-gel detection or western blot and infrared analysis.

### Protein purification

#### Purification of GST fusion proteins

Supernatant of centrifuged samples was used for purification using a 1-mL GSTrap HP column (GE Healthcare, Munich, Germany) coupled to the Äkta-Purifier FPLC system (GE Healthcare). Aliquots of the flow through fractions were analysed by SDS-PAGE and Coomassie staining. One-mL elution fractions containing the purified GST-GRF fusion proteins were pooled and dialyzed against PBS buffer (20 mM Na-phosphate, pH 7.4, 150 mM NaCl) in order to remove reduced glutathione from the elution buffer which is essential for subsequent pull-down analysis.

#### Purification of 6xHis fusion proteins

For the purification of the fusion proteins IFP-6xHis and TPK1-TEV-IFP-6xHis, *L. tarentolae* cells from nine 150 cm^2^-tissue culture flasks each containing 60 mL of non-selective expression medium were pooled and centrifuged. Cell pellets were resuspended in 10 mL standard Tris buffer supplemented with protease inhibitors (1 mM PMSF and EDTA-free protease inhibitor cocktail). Resuspended cells were sonicated and the supernatants of ultracentrifuged samples were used for purification. Proteins were purified using a 1-mL HisTrap HP column (GE Healthcare) coupled to the Äkta-Purifier FPLC system. For purification of the 6xHis-IFP-TEV-SAM1/-ACO1 and ANAC042-/ANAC059-TEV-IFP-6xHis fusion proteins, resuspended *E. coli* cell pellets were sonicated and the supernatants of ultracentrifuged samples were used. Proteins were purified using Protino Ni-IDA 150 packed columns (Macherey & Nagel) according to the instructions of the manufacturer.

### Infrared analysis of IFP fusion proteins

For in-cell detection of protein expression, IFP fusion protein-expressing *in vitro* samples (50 µL) and cells (100 µL) were transferred into the wells of clear 96-well microtiter plates with round bottom (Corning, New York, USA) followed by infrared scan at 700 nm using the Odyssey Infrared Imaging System (LI-COR Biosciences, Bad Homburg, Germany) as described before [21].

Protein samples were separated in 12% SDS-polyacrylamide gels using the Mighty Small II system (Hoefer, Massachusetts, USA) and analysed by (i) in-gel detection or (ii) immunologically. (i) For in-gel detection of the IFP moiety IFP fusion proteins were visualized after SDS-PAGE (without demounting cast protein gels) at 700 nm using the Odyssey Infrared Imaging System (LI-COR) as described before [21] and presented in grey- or red-scale. All in-gel detections were done in the presence of the PageRuler Plus Prestained Protein ladder (Fermentas, St. Leon-Rot, Germany) containing two red pre-stained marker proteins (28 and 72 kDa) visible at day light, but invisible upon excitation at 700 nm. All other marker proteins are pre-stained in blue resulting in a green fluorescent signal at 700 nm. ii) For immunological analysis SDS-PAGE-separated proteins were transferred onto Protran nitrocellulose membrane (Whatman, Kent, UK). The membrane was blocked for 1 h in blocking buffer (5% non-fat dry milk in PBS containing 0.1% Tween-20), followed by incubation for 1 h with first monoclonal mouse antibody directed against the 6xHis epitope (Santa Cruz Biotechnology, Heidelberg, Germany). Membranes were washed three times for 10 min in washing buffer (PBS containing 0.1% Tween-20) and incubated for 1 h with IRDye800CW-conjugated goat anti-mouse secondary antibody (LI-COR). All incubations were performed at room temperature and antibodies were diluted 1∶10,000 in blocking buffer. Signal intensities were analysed at 800 nm by using the Odyssey Infrared Imaging System (LI-COR).

### *In vitro* protein-protein and protein-DNA interaction assays

#### Protein-protein interaction analysis

For protein pull-down assays concentrations of purified GST and GST-GRF fusion proteins were estimated by SDS-PAGE and Coomassie staining using bovine serum albumin (BSA) as standard. *In vitro* protein-protein interaction assays were performed by using magnetic glutathione agarose beads of the MagneGST pull-down system (Promega, Mannheim, Germany). To this end, 20 µL of the magnetic particles were pretreated according to the manufacturer's instructions by incubation at room temperature for 30 min with 30% BSA. Equal amounts of purified GST and GST-GRF fusion proteins (5 µg) were immobilized on pretreated magnetic glutathione agarose beads in the presence of 0.5% Nonidet-P40 and 10% BSA by incubation at room temperature for 30 min. Immobilized GST and GST-GRF fusion proteins were incubated with equal amounts of purified IFP-6xHis and TPK1(1-79)-TEV-IFP-6xHis fusion proteins (1 µg) at room temperature for 60 min and washed five times with 400 µL of washing buffer supplemented with 0.5% Nonidet-P40. Eluted proteins were analyzed by infrared imaging (as described above) in microtiter plates followed by SDS-PAGE separation and in-gel detection.

#### Protein-DNA interaction analysis

DNA pull-down assays were carried out in three steps, i) immobilization of biotinylated double-stranded DNA molecules using a Streptavidin Mutein Matrix (Roche), ii) incubation of immobilized DNA with protein, and iii) elution of DNA-protein complex followed by infrared detection of interacting IFP fusion protein in microtiter plates, by in-gel detection and western blot analysis.

i) Biotinylated double-stranded DNA molecules (50 bp), B-100%-DNA and B-7%-DNA, were generated by annealing equimolar non-biotinylated forward oligonucleotides (2000 pmol) and their complementary 5′-biotinylated reverse oligonucleotides (2000 pmol) in a hybridization reaction for 30 min at room temperature. Forward oligonucleotide sequences for B-100%-DNA and B-7%-DNA are as follows: 5′-TAACT**GGTGCC[GT]TGACAAGACG**GCGACAGGAGTGGTGATTCCGGGCCTT-3′ and 5′-TAACT**GGTGCC[AA]TGACAAGACG**GCGACAGGAGTGGTGATTCCGGGC-CTT-3′. The two characteristic nucleotides discriminating the 100%-DNA sequence from the 7%-DNA sequence are labeled by squared parentheses. Nucleotide sequences of the ANAC042 binding sites are typed in bold and flanked by additional constant nucleotides (five nucleotides at the 5′-end and 27 nucleotides at the 3′-end). For the immobilization reaction 100 µL of centrifuged streptavidin mutein particles were pretreated according to the instructions of the manufacturer. Equal amounts of biotinylated double-stranded DNA oligonucleotides (4000 pmol each) were immobilized on equilibrated streptavidin mutein beads by incubation at room temperature for 10 min followed by washing three times with 400 µL washing buffer (see manual Streptavidin Mutein Matrix).

ii) Concentrations of purified IFP-6xHis and ANAC042-TEV-IFP-6xHis fusion proteins were estimated by SDS-PAGE and Coomassie staining using BSA as standard. Immobilized double-stranded DNA molecules were incubated with equal amounts of purified ANAC042-TEV-IFP-6xHis (experiment) and IFP-6xHis (negative control) fusion proteins (∼5 µg) at room temperature for 60 min in a total volume of 700 µL containing as competitors either 4000 pmol of non-biotinylated 7%-DNA (used for immobilized B-100%-DNA) or non-biotinylated 100%-DNA (used for immobilized B-7%-DNA). Samples were washed three times with 400 µL washing buffer (see manual Streptavidin Mutein Matrix).

iii) Interacting DNA-protein complexes were eluted from the streptavidin mutein matrix with 70 µL elution buffer (see manual Streptavidin Mutein Matrix). Proteins (50 µL) were analyzed by infrared imaging in microtiter plates, followed by SDS-PAGE separation and in-gel detection or western blot analysis. Signal intensities in microtiter plates were used for quantification of protein-DNA interactions using the Odyssey Infrared Imaging System (LI-COR).

### Enzymatic activity assays of IFP fusion proteins

Enzymatic activity assays were carried out with two cell wall degrading enzymes, endo-β-1,4-glucanase and endo-β-1,4-xylanase, after LIC of their open reading frames into the vectors LIC-pDEST-LC1 and -LC2. The resulting expression constructs, encoding for the fusion proteins 6xHis-IFP-TEV-endo-β-1,4-glucanase/-endo-β-1,4-xylanase and endo-β-1,4-glucanase-/endo-β-1,4-xylanase-TEV-IFP-6xHis, respectively, were transformed into different *E. coli* expression strains. Protein-expressing transformants were grown over-night in 3 mL medium in the presence of antibiotics. Subsequently, 2 µL of the *E. coli* cultures were transferred to petri dishes containing selection medium supplemented with 2 mM IPTG and 0.2% carboxymethylcellulose (CMC; Sigma-Aldrich) or birch wood xylan (Roth, Karlsruhe, Germany) for glucanase or xylanase activity assays, respectively. After over-night growth at 37°C, plates were stained with Congo Red to detect enzyme activities, as described by Pointing [31].

## Results

### Construction of LIC-compatible expression vectors

A set of ten LIC-compatible vectors for *in vitro* and *in vivo* protein expression were constructed by inserting PCR-generated oligonucleotide fragments into the multiple cloning sites of commercially available expression vectors. All proteins expressed from these vectors contain a TEV protease cleavable site and both, an IFP- and 6xHis-tag for protein detection. Vectors were constructed in two ways, to allow expression of fusion proteins with either the 6xHis-tag/IFP-tag/TEV cleavage site at the N-terminus (LC1 vectors; 6xHis-IFP-TEV-ProteinX), or the TEV cleavage site/IFP-tag/His-tag at the C-terminus (LC2 vectors; ProteinX-TEV-IFP-6xHis) (**Fig. 2**). All vectors described here were shown to be functional (see below).

**Figure 3:**
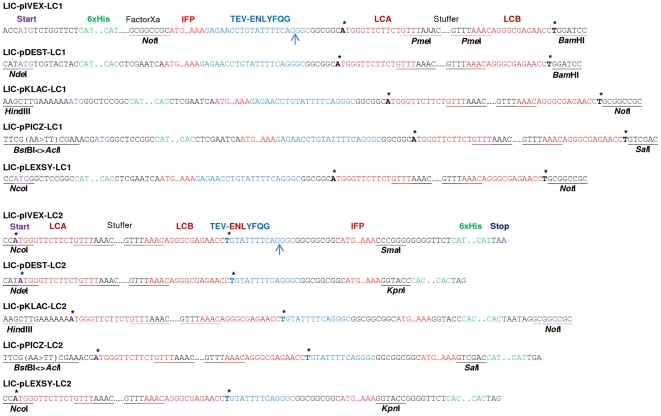
Nucleotide sequences of integrated oligonucleotide fragments. Sequences of integrated oligonucleotide fragments with features common to all LIC-LC1 and LIC-LC2 vectors are shown. Double-stranded oligonucleotides were integrated at the restriction enzyme recognition sites indicated except for PmeI which is used to eliminate the 670-bp stuffer fragment prior to the LIC process. LIC-pPICZ-LC1/-LC2 vectors were generated by inserting AclI/SalI-restricted double-stranded oligonucleotides into BstBI/SalI-digested expression vector (cutting with AclI and BstBI creates compatible 5′ overhangs), resulting in a change of the BstBI sequence (TTCGAA to TTCGTT). The asterisk on the forward strand indicates the position of adenine (corresponding to thymine on the reverse strand) required for the generation of LIC 5′ overhangs in the presence of T4 DNA polymerase and dATP. The blue arrow indicates the TEV cleavage site suitable for the removal of the marker proteins IFP and 6xHis-tag.

For *in vitro* expression, LIC-compatible vectors were derived from the pIVEX2.4d and pIVEX2.3d vectors (Roche). Vectors pDEST15 (Invitrogen), pKLAC1 (NEB), pPICZ-αA (Invitrogen) and pLEXSY-sat2 (Jena Bioscience) were made LIC-compatible for *in vivo* expression in *E. coli*, *K. lactis*, *P. pastoris* and *L. tarentolae*, respectively. Before conversion into a LIC-compatible *Pichia* expression vector an internal PmeI restriction site had to be eliminated from the pPICZ-αA vector resulting in vector pPICZ-αA-ΔPmeI. The successfully constructed LIC vectors, all verified by sequencing, were named LIC-pIVEX-LC1/-LC2, LIC-pDEST-LC1/-LC2, LIC-pKLAC-LC1/-LC2, LIC-pPICZ-LC1/-LC2 and LIC-pLEXSY-LC1/-LC2, respectively. LC1 vectors encode for amino acid sequences consisting of an N-terminal 6xHis-tag, IFP-tag and the ENLYFQG TEV cleavage site followed by the LIC site for insertion of target open reading frames. LC2 vectors encode for amino acid sequences consisting of an N-terminal LIC site for insertion of target open reading frames, followed by the ENLYFQG TEV cleavage site and the IFP- and 6xHis-tags (**Fig. 3**). To allow rapid insertion of PCR-generated open reading frames into the various vectors, the LIC sites were all made identical in the N- and C-terminal fusion vectors, respectively. Thus, PCR amplification of open reading frames for expression from ten different plasmids in four hosts and one *in vitro* transcription/translation system requires only three primers for the generation of two PCR amplicons.

To increase cloning efficiency we inserted a 670-bp long stuffer fragment, flanked by two LIC annealing sites, called LCA (15 nt) and LCB (16 nt), into each LIC-compatible vector. Each LIC site includes a PmeI restriction site (**Fig. 3**). Cutting the LIC vector with PmeI releases the stuffer fragment, leaving behind blunt-ended, linearized vector amenable for generation of 5′ single-strand overhangs by T4 DNA polymerase (see below). The TEV cleavage site is in close proximity to the native protein and allows removing the IFP- and 6xHis-tags, leaving nine amino acid residues at the N-terminus of the target proteins expressed from LC1 vectors, and ten amino acid residues at the C-terminus of the target proteins expressed from LC2 vectors (**Fig. 3**).

### Characterization of LIC-compatible expression vectors

**Figure 4:**
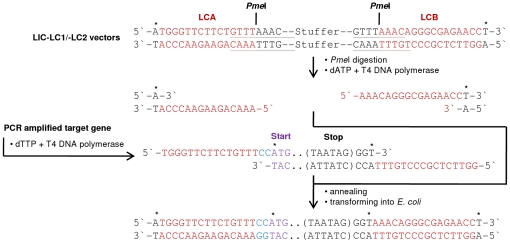
Ligation-independent cloning using LIC-IFP-compatible expression vectors. LIC vectors (LIC-LC1 and LIC-LC2) are cleaved with PmeI restriction enzyme and the released stuffer fragment (670 bp) is removed. The cleaved vector is treated with T4 DNA polymerase in the presence of dATP, whereas the PCR product (amplified open reading frame) is treated in the presence of dTTP. The asterisks indicate the position of adenine (vector) or thymine (PCR product) required for the generation of LIC-complementary 5′ overhangs. After successful annealing and transformation into *E. coli*, host-internal ligases and DNA polymerases close the vector and fill in the gaps, caused by the two additional nucleotides (CC, coloured in blue) upstream of the start codon (ATG), which are required to retain the reading frame. For LIC with LC1 vectors, PCR-amplified open reading frames contain a double stop codon (TAATAG); for LIC with LC2 vectors, open reading frames must not contain a stop codon to allow expression of ProteinX-TEV-IFP-6xHis fusion proteins. To provide the thymine moiety on the forward strand for dTTP/T4 DNA polymerase treatment, additional three nucleotides (GGT) are added directly at the 3′-end of the PCR-amplified open reading frame.

The newly generated and sequence-confirmed LIC vectors were analysed for rapid, parallel and efficient LIC by generating expression constructs encoding for different proteins derived from the plant *Arabidopsis thaliana*: the transcription factors ANAC042 (AGI code At2g43000) and ANAC059 (At3g29035), the ethylene-synthesis components SAM1 (At1g02500), ACS2 (At1g01480) and ACO1 (At2g19590), the β-galactosidases BGAL4 (At5g56870) and BGAL10 (At5g63810), and the cytosolic part (amino acids 1-79) of the membrane-located potassium channel TPK1 (At5g55630). To this end, all vectors were linearized at the LIC sites by PmeI digestion and purified from contaminating stuffer fragment, followed by T4 DNA polymerase treatment in the presence of dATP, resulting in 15 nt or 16 nt overhangs within the LCA or LCB sites (**Fig. 4**). All PCR-amplified target open reading frames (see above) with LCA and LCB complementary extensions at both ends (5′ and 3′) were treated with T4 DNA polymerase in the presence of dTTP. For each target gene two PCR products were generated using three primers in total: one forward primer containing the LCA extension and the gene-specific sequence, and two reverse primers containing the LCB extension and the gene-specific sequence with or without a stop codon, respectively. T4 DNA polymerase-treated PCR products with stop codon were used for LIC into LC1 vectors encoding for N-terminal marker proteins; PCR products without stop codon were used for LIC into LC2 vectors encoding for C-terminal marker proteins (**Fig. 4**). The complementary overhangs of the vectors and PCR products allowed highly efficient and directed LIC, independent of the size of the target open reading frames tested in this work. Cloning efficiency was proven to be 100% by plasmid isolation and restriction analysis as well as sequencing of all LIC-generated constructs after transforming into competent cells. Features of all LIC-IFP vectors (ten in total) and expression plasmids (54 in total) are summarized in **Table 1**.

**Table 1:** Features of LIC-IFP and expression vectors.

Host	Parental	LIC-IFP vector	Expression vectors/MW
	vector	(leader sequence)	
*In vitro*	pIVEX2.4d	LIC-pIVEX-LC1	LIC-pIVEX-LC1-SAM1/82.2 kDa
		(6xHis-IFP-TEV-LIC)	LIC-pIVEX-LC1-ACO1/74.3 kDa
			LIC-pIVEX-LC1-ACS2/94.7 kDa
			LIC-pIVEX-LC1-ANAC042/70.7 kDa
			LIC-pIVEX-LC1-ANAC059/75 kDa
*In vitro*	pIVEX2.3d	LIC-pIVEX-LC2	LIC-pIVEX-LC2-SAM1/81.8 kDa
		(LIC-TEV-IFP-6xHis)	LIC-pIVEX-LC2-ACO1/72.7 kDa
			LIC-pIVEX-LC2-ACS2/94.2 kDa
			LIC-pIVEX-LC2-ANAC042/70.2 kDa
			LIC-pIVEX-LC2-ANAC059/74.9 kDa
*E. coli*	pDEST15	LIC-pDEST-LC1	LIC-pDEST-LC1-SAM1/82 kDa
		(6xHis-IFP-TEV-LIC)	LIC-pDEST-LC1-ACO1/74 kDa
			LIC-pDEST-LC1-ACS2/94.3 kDa
			LIC-pDEST-LC1-ANAC042/70.3 kDa
			LIC-pDEST-LC1-ANAC059/74.6 kDa
			LIC-pDEST-LC1-BGAL4/79.8 kDa
			LIC-pDEST-LC1-BGAL10/79.8 kDa
			LIC-pDEST-LC1-Endo-β-1,4-glucanase/74.6 kDa
			LIC-pDEST-LC1-Endo- β-1,4-xylanase/62.9 kDa
*E. coli*	pDEST15	LIC-pDEST-LC2	LIC-pDEST-LC2-ANAC042/70 kDa
		(LIC-TEV-IFP-6xHis)	LIC-pDEST-LC2-ANAC059/74.3 kDa
			LIC-pDEST-LC2-BGAL4/79.5 kDa
			LIC-pDEST-LC2-BGAL10/79.5 kDa
			LIC-pDEST-LC2-Endo-β-1,4-glucanase/74.3 kDa
			LIC-pDEST-LC2-Endo- β-1,4-xylanase/62.6 kDa
*K. lactis*	pKLAC1	LIC-pKLAC-LC1	LIC-pKLAC-LC1-SAM1/81.7 kDa
		(6xHis-IFP-TEV-LIC)	LIC-pKLAC-LC1-ACO1/73.8 kDa
			LIC-pKLAC-LC1-ANAC059/74.4 kDa
*K. lactis*	pKLAC1	LIC-pKLAC-LC2	LIC-pKLAC-LC2-SAM1/81.7 kDa
		(LIC-TEV-IFP-6xHis)	LIC-pKLAC-LC2-ACO1/73.7 kDa
			LIC-pKLAC-LC2-ANAC059/74.3 kDa
*P. pastoris*	pPICZ-αA	LIC-pPICZ-LC1	LIC-pPICZ-LC1-SAM1/81.8 kDa
		(6xHis-IFP-TEV-LIC)	LIC-pPICZ-LC1-ACO1/73.8 kDa
			LIC-pPICZ-LC1-ACS2/94.1 kDa
			LIC-pPICZ-LC1-ANAC042/70.1 kDa
			LIC-pPICZ-LC1-BGAL4/79.6 kDa
			LIC-pPICZ-LC1-BGAL10/79.6 kDa
*P. pastoris*	pPICZ-αA	LIC-pPICZ-LC2	LIC-pPICZ-LC2-SAM1/81.7 kDa
		(LIC-TEV-IFP-6xHis)	LIC-pPICZ-LC2-ACO1/73.8 kDa
			LIC-pPICZ-LC2-ACS2/94.1 kDa
			LIC-pPICZ-LC2-ANAC042/70 kDa
			LIC-pPICZ-LC2-BGAL4/79.6 kDa
			LIC-pPICZ-LC2-BGAL10/79.5 kDa
*L. tarentolae*	pLEXSY-sat2	LIC-pLEXSY-LC1	LIC-pLEXSY-LC1-TPK1(1-79)/47.7 kDa
		(6xHis-IFP-TEV-LIC)	LIC-pLEXSY-LC1-ANAC042/70 kDa
			LIC-pLEXSY-LC1-BGAL4/79.6 kDa
			LIC-pLEXSY-LC1-BGAL10/79.6 kDa
*L. tarentolae*	pLEXSY-sat2	LIC-pLEXSY-LC2	LIC-pLEXSY-LC2-TPK1(1-79)/47.6 kDa
		(LIC-TEV-IFP-6xHis)	LIC-pLEXSY-LC2-SAM1/81.7 kDa
			LIC-pLEXSY-LC2-ACO1/73.7 kDa
			LIC-pLEXSY-LC2-ACS2/94.1 kDa
			LIC-pLEXSY-LC2-ANAC042/70 kDa
			LIC-pLEXSY-LC2-BGAL4/79.5 kDa
			LIC-pLEXSY-LC2-BGAL10/79.5 kDa

### Protein expression *in vitro* and *in vivo* using LIC-IFP-compatible vectors: general aspects

Protein synthesis capability was investigated after LIC of target open reading frames into the newly generated LIC-compatible vectors using a commercial *in vitro* transcription/translation kit (‘RTS 100 *E. coli* HY Kit’, derived from *E. coli* extracts) and several *in vivo* systems including various *E. coli* strains [BL21 (DE3) pLysS, BL21 (DE3) CodonPlus-RIL, Rosetta (DE3) pRARE, and BL21 Star (DE3) pRARE], *K. lactis* (GG799), *P. pastoris* (X33) and *L. tarentolae* (P10); for details see Material and Methods.

**Figure 5:**
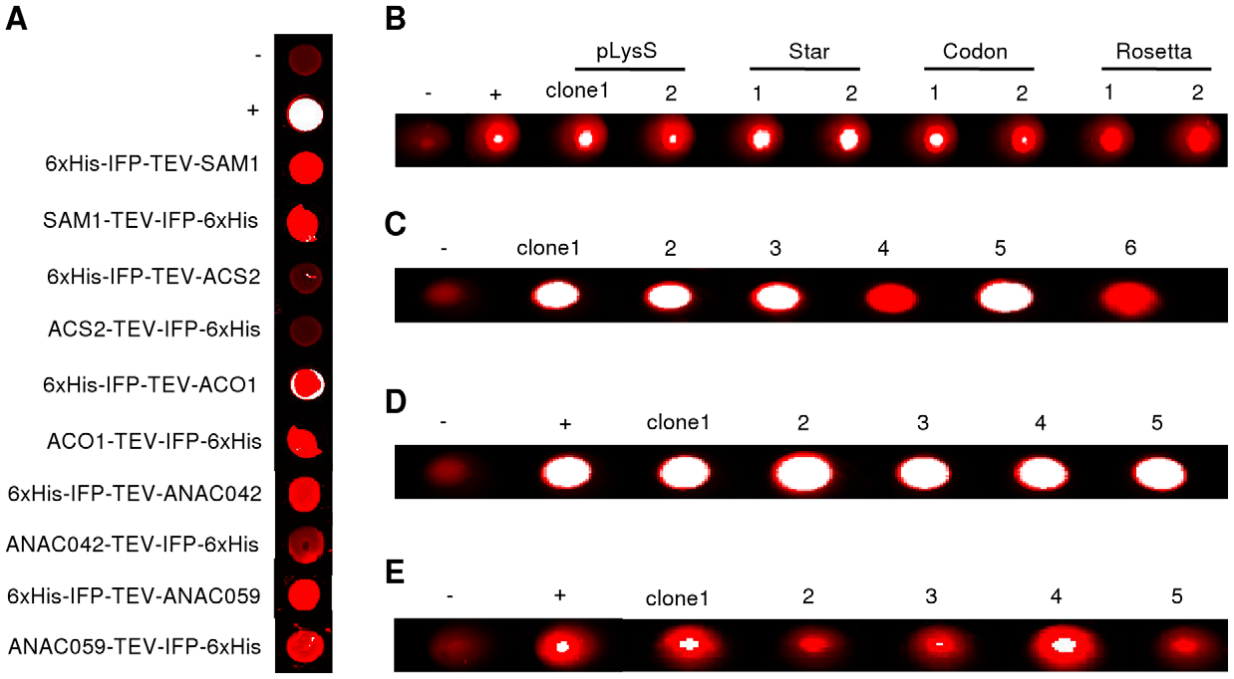
Infrared analysis of *in vitro* and *in vivo* expressed IFP fusion proteins. Infrared scanning of all samples was performed in microtiter plates using the Odyssey Infrared Imaging System from LI-COR Biosciences. (**A**) *In vitro* transcription/translation products were analysed by infrared scanning using the whole reaction mixtures. 6xHis-GFP and IFP-6xHis fusion protein-expressing samples were used as negative (-) and positive (+) controls, respectively. (**B**) In-cell detection of IFP fusion protein (6xHis-IFP-TEV-ANAC042 shown as an example) in two randomly selected clones each from the *E. coli* strains (BL21 (DE3) pLysS (‘pLysS’), BL21 Star (DE3) pRARE (‘Star’), BL21 (DE3) CodonPlus-RIL (‘Codon’), and Rosetta (DE3) pRARE (‘Rosetta’). 6xHis-GFP and IFP-6xHis fusion protein-expressing cells were used as negative (-) and positive (+) controls, respectively. (**C**), (**D**) and (**E**) In-cell detection of IFP fusion protein (SAM1-TEV-IFP-6xHis shown as an example) in randomly selected *K. lactis*, *P. pastoris* and *L. tarentolae* clones. Cell lines not expressing IPF or expressing IFP-6xHis fusion protein were used as negative (-) and positive (+) controls, respectively. No positive control was available for expression in *K. lactis*. Note that strong infrared signal appears white in the digital images.

Expression of fusion proteins *in vitro* or *in vivo* was analysed by infrared scanning of the IFP moiety and/or by immunological detection of the 6xHis-tag. In case of *in vitro* transcription/translation, aliquots of crude protein extracts were transferred to microtiter plates for infrared excitation (**Fig. 5**). In the case of *in vivo* protein expression, intact cells were transferred to the microtiter plates and analysed by infrared imaging (**Fig. 5**). As reported before for protein production in *Leishmania* [21] this allows pre-selection of well expressing cell lines at an early stage of the expression pipeline. As infrared imaging of microtiter plates does not allow distinguishing between full-length and truncated IFP-containing proteins we additionally separated crude protein extracts obtained by *in vitro* expression as well as pellet and supernatant fractions of disrupted and ultracentrifuged *in vivo* samples by SDS-PAGE; after gel electrophoresis the IFP moiety of IFP fusion proteins was detected by in-gel infrared imaging (estimated detection limit ∼100 ng/lane; data not shown), and the 6xHis-tag was detected by western blot. We analyzed insoluble pellet (protein precipitates, inclusion bodies) and soluble protein of the supernatant after ultracentrifugation to compare solubility of expressed proteins in the different expression systems.

### *In vitro* protein expression of IFP fusion proteins using LIC-pIVEX-LC1/-LC2 vectors

**Figure 6:**
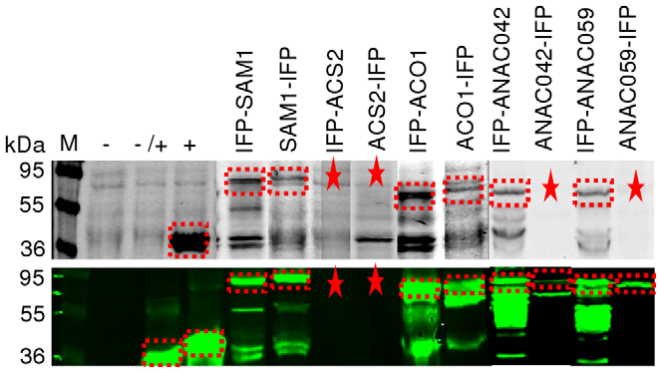
LIC cloning and expression of IFP fusion proteins *in vitro*. *In vitro* expressed IFP fusion proteins were separated by SDS-PAGE and analysed by in-gel detection directly in the cast gel at 700 nm (upper panel) followed by western transfer and immunological detection at 800 nm (lower panel). Plasmid-free translation extract (-), as well as samples expressing 6xHis-GFP fusion protein (-/+) or IFP-6xHis fusion protein (+) were used as controls. Detected or expected protein bands are labelled by dashed frames or asterisks, respectively. M, molecular mass marker (kDa).

Protein synthesis employing the newly generated LIC-pIVEX-LC1/-LC2 *in vitro* expression vectors was investigated using five different target proteins, i.e. ANAC042, ANAC059, SAM1, ACS2 and ACO1, fused in both orientations (N- and C-terminal) to the marker proteins IFP and 6xHis-tag, resulting in a total of ten fusion proteins. Six or eight of the ten IFP fusion proteins were detected by in-gel imaging (**Fig. 6**, upper panel; 6xHis-IFP-TEV-SAM1/-ACO1/-ANAC042/-ANAC059 and SAM1-/ACO1-TEV-IFP-6xHis) or western blot (**Fig. 6**, lower panel; 6xHis-IFP-TEV-SAM1/-ACO1/-ANAC042/-ANAC059 and SAM1-/ACO1-/ANAC042-/ANAC059-TEV-IFP-6xHis) indicating that in-gel detection in combination with immunological detection is a powerful tool for the analysis of proteins fused to the marker proteins used in this work. ACS2 was the only target protein that was not expressed or could not be detected, independent of its orientation relative to the fused marker proteins. Of note, infrared imaging generated clearly detectable signals only in the presence of IFP fusion proteins, with low or no background signal (**Fig. 6**). An important observation was that *in vitro* protein synthesis was almost completely blocked when biliverdin hydrochloride (dissolved in dimethyl sulfoxide, DMSO) was present in the transcription/translation mix. Therefore, for efficient protein expression and development of infrared signal, biliverdin hydrochloride had to be added after finishing *in vitro* protein synthesis.

### Protein expression in *E. coli* using LIC-pDEST-LC1/-LC2 vectors

**Figure 7:**
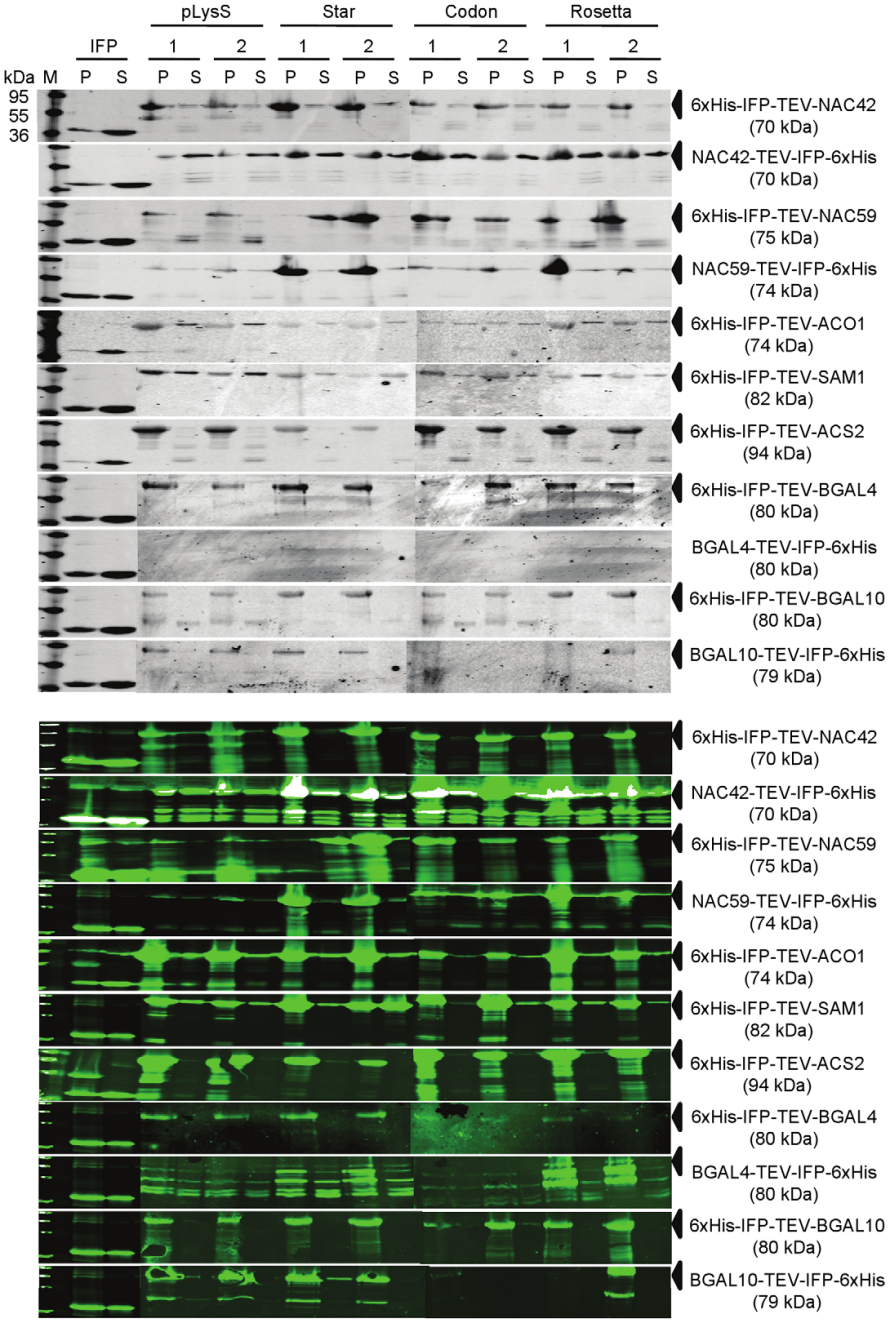
Expression of IFP fusion proteins in *E. coli*. Protein extracts obtained from IFP fusion protein-expressing *E. coli* strains BL21 (DE3) pLysS (‘pLysS’), BL21 Star (DE3) pRARE (‘Star’), BL21 (DE3) CodonPlus-RIL (‘Codon’), and Rosetta (DE3) pRARE (‘Rosetta’) were separated by SDS-PAGE and analysed by in-gel infrared imaging at 700 nm to detect IFP moieties (upper panel), followed by western transfer and immunological detection at 800 nm (using monoclonal mouse antibody directed against the 6xHis epitope; lower panel). IFP-6xHis fusion protein-expressing cells were used as positive control. Supernatant (S) and pellet (P) fractions of disrupted cells were analyzed after ultracentrifugation. M, molecular mass marker (kDa). Arrows indicate expected proteins.

Protein synthesis capability of the *E. coli* LIC-pDEST-LC1/-LC2 expression vectors was investigated using four different *E. coli* expression strains and seven different target proteins, i.e. ANAC042, ANAC059, SAM1, ACS2, ACO1, BGAL4 and BGAL10. Except for SAM1, ACS2 and ACO1 all proteins were fused in both orientations (N- and C-terminal) to the IFP- and 6xHis-tags, resulting in a total of eleven fusion proteins. Ten or eleven of the IFP fusion proteins were detected by in-gel infrared imaging (**Fig. 7**, upper panel) or immunologically (**Fig. 7**, lower panel). BGAL4-TEV-IFP-6xHis was the only protein that could not be detected by in-gel detection. As for *in vitro* expression (and expression in other organisms, see below), clear infrared signal was only detected in the presence of IFP fusion proteins. BGAL4 and BGAL10 exclusively accumulated in the insoluble fraction. Most other fusion proteins (ANAC042, ANAC059, SAM1, ACS2 and ACO1) had greater proportions of insoluble protein but were also present as soluble proteins detectable by in-gel infrared imaging and western blot analysis. Although it is known that expression at lower temperatures may increase the proportion of soluble protein [12] an optimization of the expression parameters was not attempted in this study as this greatly depends on the expressed protein. Furthermore, the solubility of proteins expressed in *E. coli* may be increased by selecting expression strains with different characteristics, e.g. those supporting the formation of disulfide bonds in their cytoplasm (Rosetta gami 2 (DE3) pLacI; Merck) or by using an improved BL21 host strain for soluble protein expression (SoluBL21; AMS Biotechnology, Abingdon, UK) [32]. Whilst *in vitro* expression of IFP or IFP fusion proteins has to be carried out in the absence of biliverdin hydrochloride, protein expression in *E. coli* is seemingly not affected by its presence. Instead of biliverdin hydrochloride, also hemin can be fed, which upon co-expression of a cyanobacterial heme oxygenase (HO-1) is converted to biliverdin in *E. coli* (data not shown, and [20]). Using hemin instead of biliverdin hydrochloride may reduce costs, however, for co-expression using two expression vectors a third antibiotic selection marker (for maintenance of the HO-1 expression vector) is then required. Thus, for both, IFP fusion protein screening in *E. coli* in small expression volumes and up-scaling of protein expression in larger volumes we recommend to express IFP fusion proteins in medium lacking biliverdin hydrochloride, followed by disruption of harvested cells in lysis buffer that contains biliverdin hydrochloride.

### Protein expression in *K. lactis*, *P. pastoris* and *L. tarentolae* using LIC-pKLAC-LC1/-LC2, LIC-pPICZ-LC1/-LC2 and LIC-pLEXSY-LC1/-LC2 vectors

**Figure 8:**
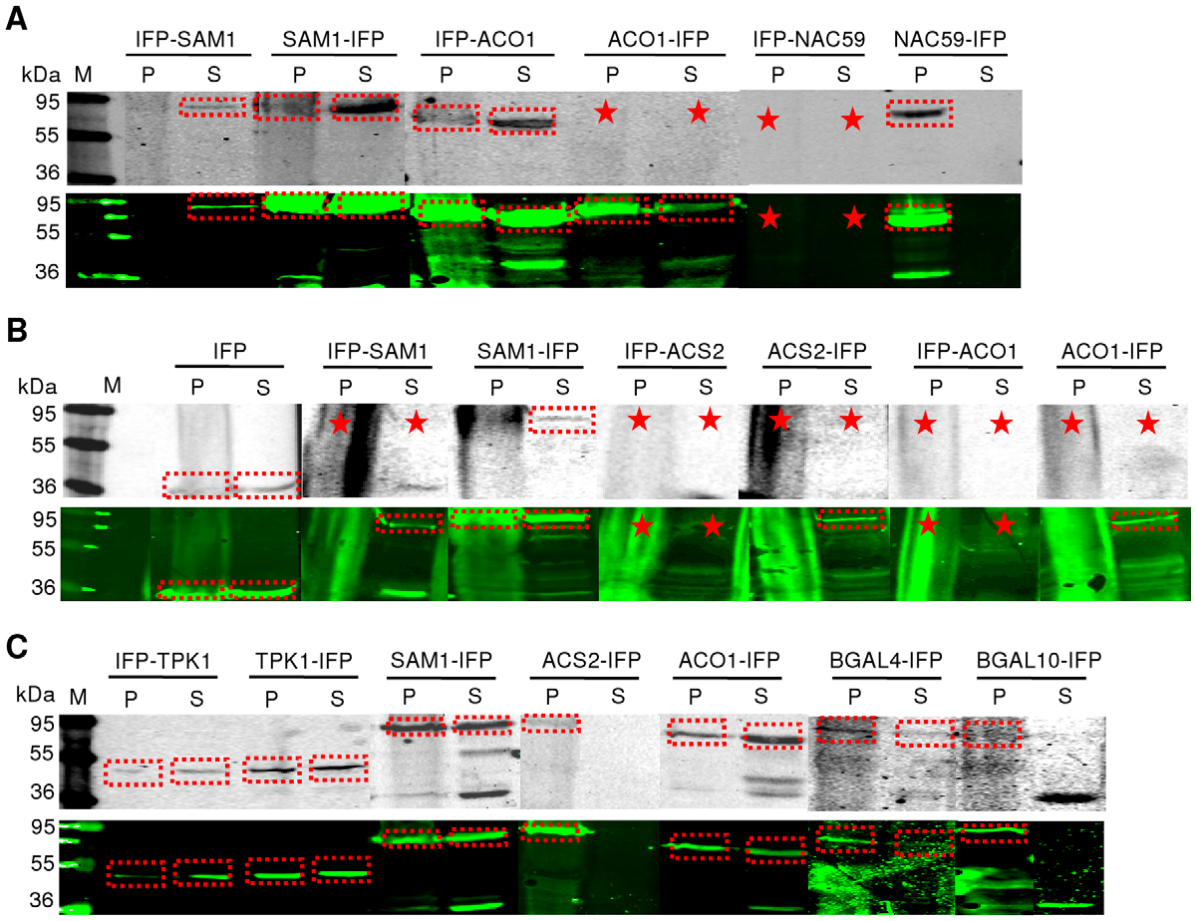
Expression of IFP fusion proteins in eukaryotic cells. Proteins extracted from (**A**) *Kluyveromyces lactis*, (**B**) *Pichia pastoris* and (**C**) *Leishmania tarentolae* were separated by SDS-PAGE and analysed by in-gel infrared imaging at 700 nm to detect IFP moieties (upper panels), followed by western transfer and immunological detection at 800 nm (using monoclonal mouse antibody directed against the 6xHis epitope; lower panels). Supernatant (S) and pellet (P) fractions of disrupted cells were used for analysis after ultracentrifugation. Detected or expected protein bands are labelled by dashed frames or asterisks, respectively. IFP fusion proteins present in pellet fractions from *P. pastoris* did not separate as distinct bands. M, molecular mass marker (kDa).

Protein synthesis capability of the newly generated expression vectors LIC-pKLAC-LC1/-LC2 (*K. lactis*), LIC-pPICZ-LC1/-LC2 (*P. pastoris*) and LIC-pLEXSY-LC1/-LC2 (*L. tarentolae*) was investigated by expressing various numbers of proteins from the following collection: ACO1, ACS2, ANAC042, ANAC059, BGAL4, BGAL10, TPK1(1-79), and SAM1. Two proteins (BGAL4 and BGAL10) contained C-terminal fusions to IFP and the 6xHis-tag, and the remaining six proteins harboured fusions to both tags at their N- or C-terminus. **Figure 8** shows the results obtained by in-gel imaging (upper panels in A to C) and western blot analysis (lower panels). In the case of *K. lactis* most proteins were detected by in-gel imaging and immunologically (**Fig. 8A**). Whilst 6xHis-IFP-TEV-SAM1 accumulated exclusively in the soluble fraction and ANAC059-TEV-IFP-6xHis in the insoluble fraction, the remaining proteins (6xHis-IFP-TEV-ACO1 and SAM1-/ACO1-TEV-IFP-6xHis) were present in the soluble and insoluble fractions. Several proteins were also successfully expressed in *P. pastoris* (**Fig. 8B**). However, 6xHis-IFP-TEV-ACS2/-ACO1 proteins (**Fig. 8B**) and the four β-galactosidase fusions (6xHis-IFP-TEV-BGAL4/-BGAL10 and BGAL4-/BGAL10-TEV-IFP-6xHis; not shown) were not detectably produced by this organism. In *L. tarentolae*, seven IFP fusion proteins were expressed, i.e. 6xHis-IFP-TEV-TPK1(1-79) and TPK1(1-79)-/SAM1-/ACS2-/ACO1-/BGAL4-/BGAL10-TEV-IFP-6xHis, and detected by in-gel imaging (**Fig. 8C**, upper panel) and western blot (**Fig. 8C**, lower panel). ANAC042 fused to the marker proteins in both orientations as well as 6xHis-IFP-TEV-BGAL4- and -BGAL10 proteins were not visibly expressed in *Leishmania* (not shown).

Taken together, in-gel detection resulted in clearly visible infrared signals in all expression systems in the presence of IFP or IFP fusion proteins with biliverdin hydrochloride or hemin as co-factor. Truncated IFP fusion proteins were occasionally observed (e.g. **Fig. 6**, IFP-SAM1 or IFP-ACO1) and were likely due to protein instability, premature termination of protein synthesis or translation initiation at internal ribosome binding sites. However, if wanted, this can be optimized by e.g. the addition of protein stabilizing components (e.g. glycerin) or changing expression parameters (expression time and temperature, concentration of inducers, changing expression strains), using additional protease inhibitors, or optimizing protein-coding sequences by gene synthesis to adapt codon usage, modify secondary RNA structures and avoid internal ribosome binding sites. For protein expression in *Leishmania* we used media containing hemin. However, in *K. lactis* and *P. pastoris*, the continous presence of hemin had a negative effect on protein formation and/or the intensity of the IFP signal. In these cases we therefore added hemin only two hours before cell culture and protein expression was stopped. The eukaryotic expression systems used in this report are able to express functional IFP or IFP fusion proteins with hemin as co-factor, indicating that hemin can be catabolized by *Leishmania* (as previously shown) [21], *K. lactis* and *P. pastoris*.

### Purification of IFP fusion proteins with TEV-accessible cleavage sites

**Figure 9:**
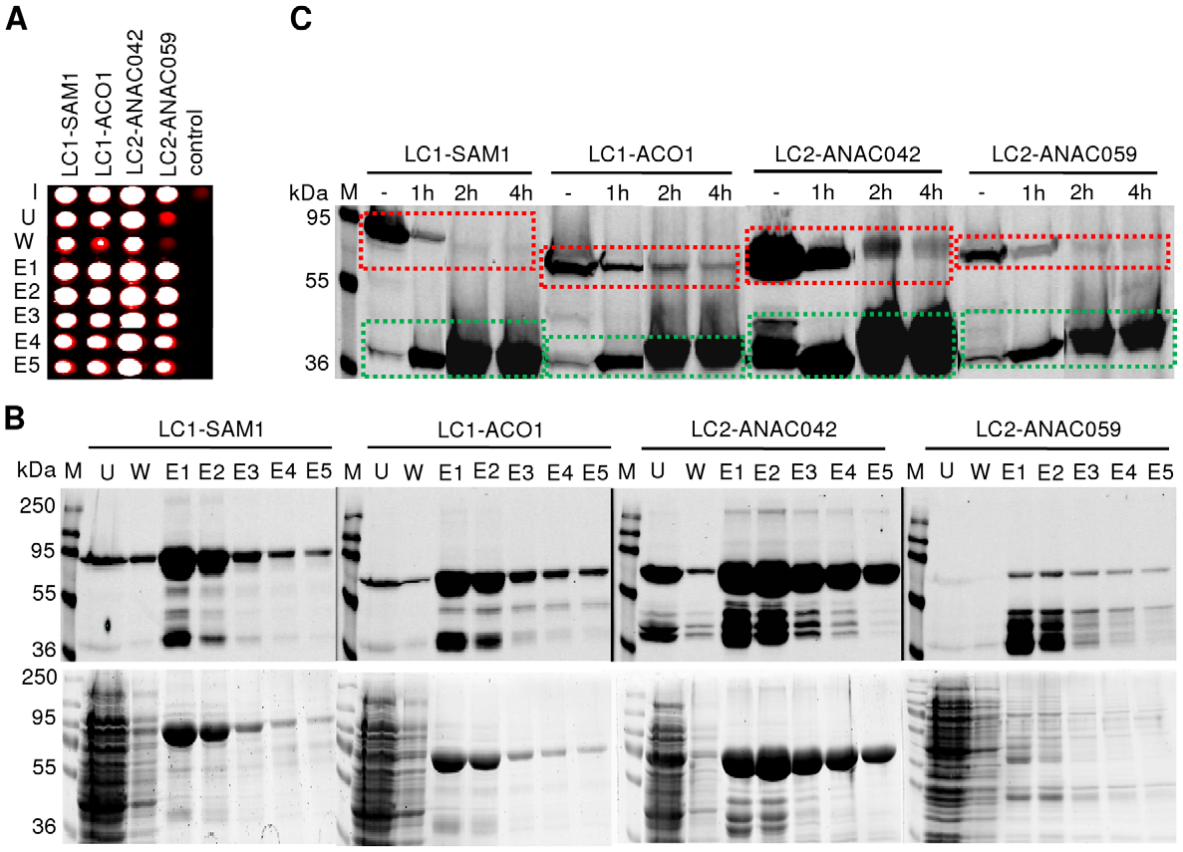
Purification of IFP fusion proteins with accessible TEV cleavage sites. Fusion proteins, i.e. 6xHis-IFP-TEV-SAM1/-ACO1 (LC1-SAM1, 82 kDa and LC1-ACO1, 74 kDa) and ANAC042-/ANAC059-TEV-IFP-6xHis (LC2-ANAC042, 70 kDa and LC2-ANAC059, 74 kDa) expressed in *E. coli* were either used for affinity purification with Protino NI-IDA 150 columns after ultracentrifugation (**A, B**) or (**C**) TEV cleavage experiments directly in crude extracts. (**A, B**) Input (row and lane ‘I’) and 50-µL aliquots of the fractions ‘unbound’ (row and lane ‘U’), ‘wash’ (row and lane ‘W’) and ‘elution’ (rows and lanes ‘E1’ to ‘E5’) were analyzed by infrared imaging in (**A**) microtiter plates (control corresponds to *E. coli* cells expressing GST alone; note that strong infrared signal appears white in the digital image) or by in-gel detection and Coomassie staining (**B**, upper and lower panel) after SDS-PAGE separation. (**C**) Aliquots of untreated (lane ‘-’) or 1 h, 2 h and 4 h TEV protease-treated crude extracts (lanes ‘1 h’, ‘2 h’ and ‘4 h’) were analysed by in-gel detection after SDS-PAGE separation. Dashed lines indicate the time-dependent decrease (red dashed boxes) or increase (green dashed boxes) of full-length IFP fusion proteins or released IFP moieties, respectively. M, molecular mass marker (kDa).

We next tested the functionality of the 6xHis and TEV leader sequences fused in both orientations (N- and C-terminal) to various IFP fusion proteins, i.e. 6xHis-IFP-TEV-SAM1/-ACO1 and ANAC042-/ANAC059-TEV-IFP-6xHis, expressed in *E. coli*. Functionality of the 6xHis tag was analysed by affinity purification of all proteins under non-denaturing conditions, using Protino NI-IDA 150 columns. Purification was accompanied by infrared analysis of 50-µL aliquots of each fraction in microtiter plates (**Fig. 9A**), followed by in-gel detection of IFP fusion proteins after SDS-PAGE (**Fig. 9B**, upper panel) or Coomassie staining of protein bands (**Fig. 9B**, lower panel). **Figure 9** clearly demonstrates that IFP fusion proteins can be easily purified by virtue of their 6xHis tag, irrespective of whether IFP is fused to target proteins via their N- or C-terminus. Importantly, purification of IFP fusion proteins can be easily monitored either during or at the end of the purification process by infrared analysis in microtiter plates or by in-gel detection after SDS-PAGE separation. However, only in-gel imaging or western blot analysis can reveal whether the infrared signal detected in microtiter plates is derived from full-length IFP fusion proteins or truncated IFP molecules.

We also tested the functionality of the TEV cleavage site. To this end, cells were disrupted and IFP fusion proteins were treated with TEV protease. Time-dependent changes of infrared signal intensity were monitored by in-gel detection after SDS-PAGE of TEV-treated proteins. Whilst infrared signal intensities of full-length IFP fusion proteins decreased over time, a clear increase of infrared signal intensity was observed for the released IFP moiety within 4h for all proteins analyzed (**Fig. 9C**), clearly demonstrating the functionality of the TEV cleavage site in IFP fusion proteins.

### Monitoring protein-protein interactions based on IFP fusions

We reasoned that IFP might not only be useful as an easy-to-handle reporter for protein expression *in vitro* and *in vivo* but may also be beneficial in other experiments where proteins need to be monitored. We therefore first tested whether IFP can be used as a reporter in protein-protein interaction studies and then analyzed whether it could also be employed to monitor protein-DNA interactions (see below).

To investigate the potential value of IFP as a reporter for protein-protein interactions, we expressed GRF1 - GRF6 proteins from *Arabidopsis thaliana* as GST fusions and tested whether they interact with the potassium channel TPK1 in pull-down assays. We have previously shown by yeast two-hybrid analysis that the amino-terminal segment of TPK1 (encompassing amino acids 1-79) interacts with GRF1 - GRF6 [33]. Except for two interactions (TPK1 with GRF2 and GRF4), all other interactions were verified by either pull-down analysis based on GST fusions (interaction of TPK1(1-79) with GRF1 and GRF6) [29] or Bimolecular Fluorescence Complementation (BiFC) experiments (interaction of TPK1(1-79) with GRF3, 5 and 6) [33]. Here, we demonstrate physical interactions of TPK1(1-79) expressed as TPK1(1-79)-TEV-IFP-6xHis fusion protein with all GRFs tested (GRF1 – GRF6).

**Figure 10:**
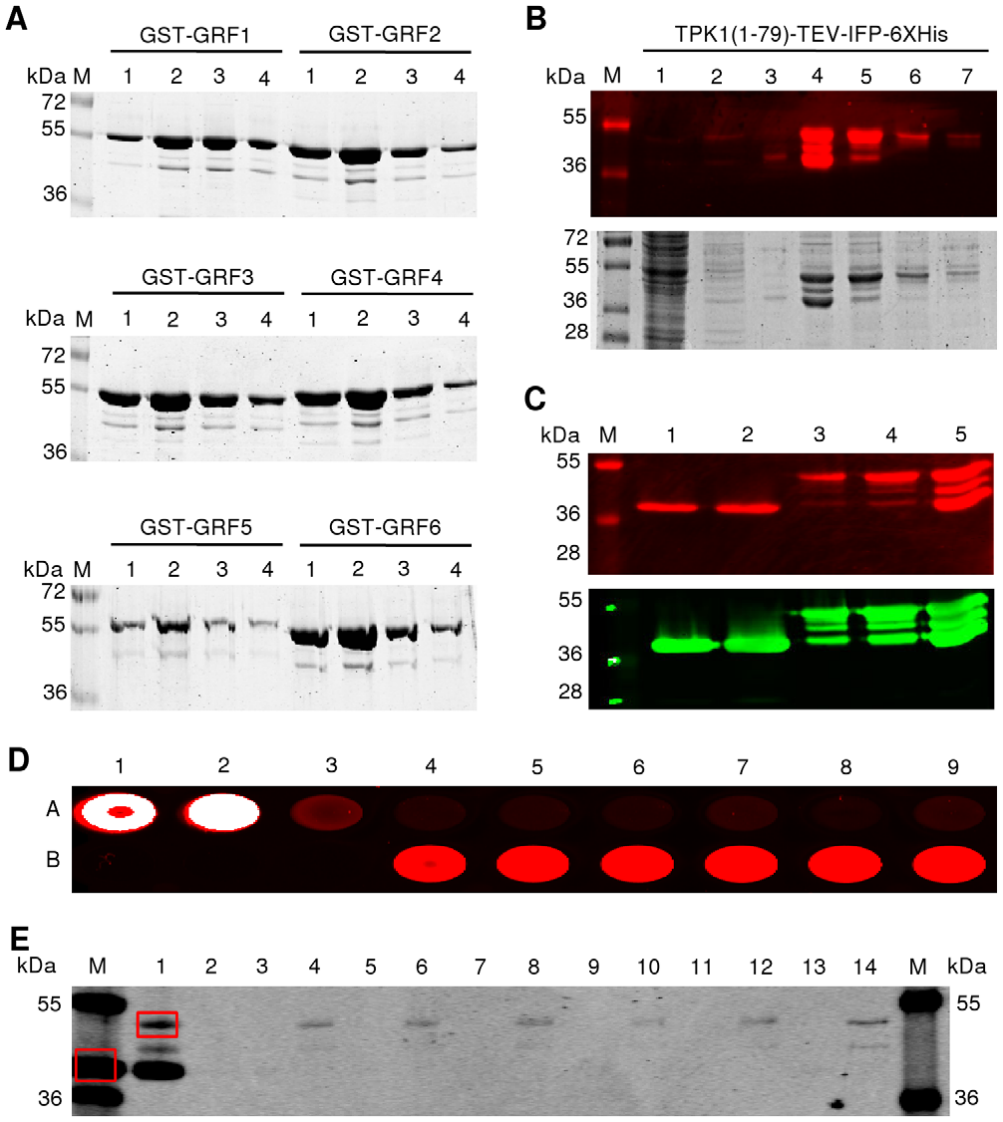
Protein-protein interaction analysis based on GST-pull down, using IFP reporter. (**A**) Fusion proteins GST-GRF1 (58 kDa), -GRF2 (57 kDa), -GRF3 (56 kDa), -GRF4 (58 kDa), -GRF5 (58 kDa) and -GRF6 (56 kDa) were expressed in *E. coli*, affinity-purified and analysed (1-mL fractions) by SDS-PAGE and Coomassie staining. (**B**) TPK1(1-79)-TEV-IFP-6xHis fusion protein (48 kDa) expressed in *L. tarentolae* and purified by affinity chromatography. One-mL fractions ‘unbound’ (lane 1), ‘washed’ (lanes 2 and 3) and ‘elution’ (lanes 4-7) were separated by SDS-PAGE and infrared-scanned (upper panel) followed by Coomassie staining (lower panel). (**C**) Signal intensities of purified fusion proteins IFP-6xHis (lane 1: 3 µL; lane 2: 5 µL) and TPK1(1-79)-TEV-IFP-6xHis (lane 3: 3 µL; lane 4: 5 µL; lane 5: 10 µL), isolated from *L. tarentolae*, were analysed by infrared-scanning after SDS-PAGE (upper panel), followed by western blot analysis (lower panel). (**D**) GST-fusion proteins immobilized on glutathione agarose beads and incubated with purified TPK1(1-79)-TEV-IFP-6xHis fusion protein. After elution, fractions were scanned at 700 nm (microtiter plate). A1: IFP-6xHis input. A2: TPK1(1-79)-TEV-IFP-6xHis input. A3: negative control with GST immobilized on glutathione agarose beads + TPK1(1-79)-TEV-IFP-6xHis. A4/5/6/7/8/9: negative controls with GST-GRF1/2/3/4/5/6 immobilized on glutathione agarose beads + IFP-6xHis. B1/2/3: empty wells. B4/5/6/7/8/9: GST-GRF1/2/3/4/5/6 immobilized on glutathione agarose beads + TPK1(1-79)-TEV-IFP-6xHis. White representation in the digital image indicates strong infrared signal. (**E**) After infrared-scanning in microtiter plates (see D) samples were separated by SDS-PAGE and scanned at 700 nm. Lane ‘M’, molecular mass marker (in kDa) supplemented with IFP-6xHis protein (red square). This protein was omitted from the marker in the last lane. Lane 1: TPK1(1-79)-TEV-IFP-6xHis (red square) input. Lane 2: negative control with GST immobilized on glutathione agarose beads + TPK1(1-79)-TEV-IFP-6xHis. Lanes 3/5/7/9/11/13: additional negative controls with GST-GRF1/2/3/4/5/6 immobilized on glutathione agarose beads + IFP-6xHis. Lanes 4/6/8/10/12/14: experiments with GST-GRF1/2/3/4/5/6 immobilized on glutathione agarose beads + TPK1(1-79)-TEV-IFP-6xHis.

GST-GRF fusion proteins were expressed in *E. coli* and affinity-purified by Äkta-FPLC using a 1-mL GSTrap column. Ten µL of the elution steps (1-mL fractions) were separated by SDS-PAGE and protein bands were visualized by Coomassie staining (**Fig. 10A**). Full-length GST-GRF fusion proteins in the range of 1–10 µg per mL of *E. coli* expression culture were obtained. Infrared-functional TPK1(1-79)-TEV-IFP-6xHis fusion protein was purified from *L. tarentolae* by Äkta-FPLC using a 1-mL HisTrap HP column. Ten µL of the collected unbound flow-through, as well as flow-through of wash and elution steps (1-mL fractions) were separated by SDS-PAGE and analyzed by in-gel detection for infrared fluorescence directly in the cast protein gel (**Fig. 10B**, upper panel) followed by Coomassie staining for visualization of all proteins (**Fig. 10B**, lower panel). The infrared images (**Fig. 10B and C**) demonstrate that both fusion proteins, TPK1(1-79)-TEV-IFP-6xHis and IFP-6xHis, are expressed and detected as full-length proteins in the gel when excited at 700 nm.

Concentrations of the purified proteins (GST, GST-GRFs, TPK1(1-79)-TEV-IFP-6xHis and IFP-6xHis) were determined by SDS-PAGE separation and Coomassie staining in the presence of BSA standard (data not shown) before interaction analysis was started. Subsequently, for the co-affinity purification experiments equal amounts of GST and GST-GRFs were immobilized to glutathione agarose beads and incubated with equal amounts of purified TPK1(1-79)-TEV-IFP-6xHis or IFP-6xHis in different combinations: (i) GST immobilized to the beads and incubated with TPK1(1-79)-TEV-IFP-6xHis fusion protein was used as negative control in order to eliminate unspecific interactions of the TPK1(1-79)-IFP-6xHis fusion protein with the beads or with the GST part of the GST-GRF fusion proteins. (ii) As a second negative control, to avoid unspecific binding of IFP-6xHis protein to the beads or to the GST part of GST-GRF fusion proteins, we incubated bead-immobilized GST with IFP-6xHis protein. Co-affinity purifications were done in the presence of BSA as competing protein, and under stringent conditions in the presence of the detergent Nonidet-P40. Co-purified proteins were specifically eluted from the beads using reduced glutathione and analyzed in microtiter plates followed by in-gel detection (**Fig. 10D and E**). **Figure 10D** (wells A3 to A9) clearly demonstrates that there is neither (or only weak) unspecific binding of TPK1(1-79)-TEV-IFP-6xHis to immobilized GST nor of IFP-6xHis to immobilized GST-GRFs. In contrast strong infrared signals can be seen in wells B4 to B9 indicating physical interactions between GRF proteins with the TPK1(1-79) moiety of the TPK1(1-79)-TEV-IFP-6xHis fusion protein. This result was verified by in-gel detection (**Fig. 10E**) where full-length TPK1(1-79)-TEV-IFP-6xHis fusion protein can only be seen in lanes where interacting proteins were specifically eluted from the beads.

### Monitoring of protein-DNA interactions based on IFP fusions

Protein-DNA interactions play an important role in many biological processes, e.g. DNA packaging, transcriptional regulation and DNA replication, besides others. Transcription factors (TFs) are proteins that interact in a sequence-specific manner with *cis*-elements in promoters of target genes. Discovering binding sites for TFs and the promoters to which they bind is of prime importance for the understanding of gene regulatory networks they control. Here we intended to test whether TF-IFP fusion proteins can be used to demonstrate binding of TFs to *cis*-elements *in vitro*, using the *Arabidopsis thaliana* NAC TF ANAC042 as a test protein. We have recently shown that ANAC042 is a key regulator of longevity in *Arabidopsis*. ANAC042 binds to a bipartite *cis*-regulatory element, as shown by *in vitro* binding site selection assay (Wu *et al*., manuscript in preparation). Through mutational analysis we identified nucleotide positions within the ANAC042 binding sites important for binding of the TF. Here, to establish an IFP-based DNA-protein interaction assay, we chose both, the wild-type *cis*-element and a mutant version of it that in previous *in vitro* experiments (using CELD-based binding-site selection assay) [34] displayed only 7% binding affinity (B-7%-DNA) compared to the wild-type sequence (B-100%-DNA).

**Figure 11:**
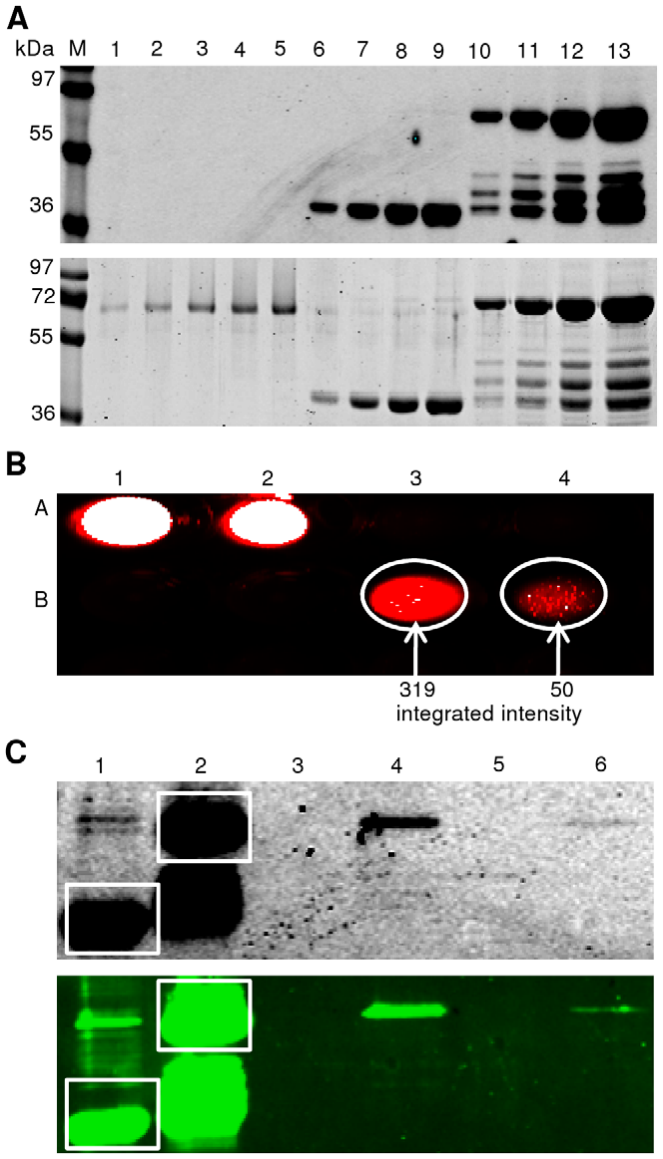
Protein-DNA interaction analysis based on IFP fusions. (**A**) Fusion proteins ANAC042-TEV-IFP-6xHis (70 kDa) and IFP-6xHis (36 kDa) affinity-purified from *E. coli*. Two elution fractions (2×250 µL) containing the purified proteins were pooled and analysed after SDS-PAGE by in-gel detection (top) and Coomassie staining (bottom) (lanes 10–13: ANAC042-TEV-IFP-6xHis, 5, 10, 15, and 20 µL; lanes 6 - 9: IFP-6xHis, 2, 5, 8 and 10 µL). BSA served as standard to estimate protein amounts (lanes 1–5: 100/250/500/750/1000 ng). Equal amounts of both proteins (∼5 µg) were used for protein-DNA interaction analysis. M, molecular mass marker (kDa). (**B**) Biotinylated dsDNA was immobilized on streptavidin mutein particles and incubated with ANAC042-TEV-IFP-6xHis protein. After elution, fractions were scanned at 700 nm in the wells of a microtiter plate (strong infrared signal appears white in the digital image). A1: IFP-6xHis input. A2: ANAC042-TEV-IFP-6xHis input. A3: negative control; B-100%-DNA immobilized on streptavidin mutein particles + IFP-6xHis in the presence of non-biotinylated 7%-DNA. A4: negative control; B-7%-DNA immobilized on streptavidin mutein particles + IFP-6xHis, in the presence of non-biotinylated 100%-DNA. B1/2: empty wells. B3/4: experiments with B-100%-DNA and B-7%-DNA immobilized on streptavidin mutein beads + ANAC042-TEV-IFP-6xHis incubated in the presence of non-biotinylated 7%- and 100%-DNA, respectively. Areas of the infrared signals were marked (white circles) and integrated signal intensities were calculated (B3 = 319, and B4 = 50). (**C**) After infrared-scanning in microtiter plates (see B) samples were separated by SDS-PAGE and scanned at 700 nm (top) followed by western blot analysis (bottom). Lane 1: IFP-6xHis input (white square). Lane 2: ANAC042-TEV-IFP-6xHis input (white square). Lane 3: negative control with B-100%-DNA immobilized on streptavidin mutein particles + IFP-6xHis, in the presence of non-biotinylated 7%-DNA. Lane 4: experiment with B-100%-DNA immobilized on streptavidin mutein particles + ANAC042-TEV-IFP-6xHis, in the presence of non-biotinylated 7%-DNA. Lane 5: negative control with B-7%-DNA immobilized on streptavidin mutein particles + IFP-6xHis, in the presence of non-biotinylated 100%-DNA. Lane 6: experiment with B-7%-DNA immobilized on streptavidin mutein particles + ANAC042-TEV-IFP-6xHis, in the presence of non-biotinylated 100%-DNA.

We expressed ANAC042-TEV-IFP-6xHis and IFP-6xHis fusion proteins in *E. coli.* After purification using Protino NI-IDA 150 columns (Macherey & Nagel) infrared-functional proteins were detected by infrared imaging after SDS-PAGE (**Fig. 11A**, upper panel). BSA standard used for quantification was only visible after Coomassie staining; calibration revealed purified proteins to be in the range of 1-10 µg per mL of *E. coli* culture (**Fig. 11A**, lower panel). For protein-DNA interaction experiments equal amounts (∼5 µg) of ANAC042-TEV-IFP-6xHis and IFP-6xHis fusion proteins were incubated with biotinylated DNA molecules (B-100%-DNA or B-7%-DNA) immobilized on streptavidin mutein particles. Control and experimental settings were as follows: (i) Beads with immobilized B-100%-DNA or B-7%-DNA were incubated with IFP-6xHis protein and used as negative controls; this treatment was expected to minimize unspecific interaction of the ANAC042-TEV-IFP-6xHis fusion protein with the beads or the immobilized DNA molecules. ii) Beads with immobilized B-100%-DNA or B-7%-DNA were incubated with ANAC042-TEV-IFP-6His fusion protein and used as experiments. All protein-DNA interactions were carried out in the presence of competing DNA: particles with immobilized B-100%-DNA or B-7%-DNA were incubated with non-biotinylated 7%-DNA or 100%-DNA, respectively, for incubation with IFP-6xHis (negative control) and ANAC042-TEV-IFP-6xHis (experiment) proteins. Interacting DNA protein complexes were specifically eluted from the beads and analyzed in microtiter plates followed by in-gel detection and western blot analysis (**Fig. 11B and C**, upper and lower panel). **Figure 11B** (positions A3 and A4 of a microtiter plate) clearly demonstrates the absence of unspecific binding of IFP-6xHis fusion protein to immobilized B-100%-DNA or B-7%-DNA. In contrast, strong binding to B-100%-DNA was observed for ANAC042-TEV-IFP-6xHis protein (position B3), whereas binding of the IFP-labeled transcription factor to B-7%-DNA was weak (position B4). Quantitative analysis using the *in silico* labeling and quantification tool of the Odyssey Infrared Imaging System (LI-COR) revealed integrated signal intensities of 319 and 50, respectively, when ANAC042-TEV-IFP-6xHis fusion protein was incubated with immobilized B-100%-DNA and B-7%-DNA (**Fig. 11B**). This result is therefore close to the data obtained independently with the CELD-based binding site selection assay (Wu *et al*., manuscript in preparation). The B-100%-DNA and B-7%-DNA differ by only two base-pairs (along a stretch of 18 base-pairs in total). According to the results obtained before and observed in this report (presented as integrated signal intensities in **Figure 11B**) a change from GT (in B-100%-DNA) to AA (in B-7%-DNA) strongly reduces interaction with the ANAC042 transcription factor. Thus, results obtained with the IFP-based DNA pull-down assay favorably compare with protein-DNA interaction data obtained using independent experimental setups.

### Enzymatic activity assays using IFP fusion proteins

**Figure 12:**
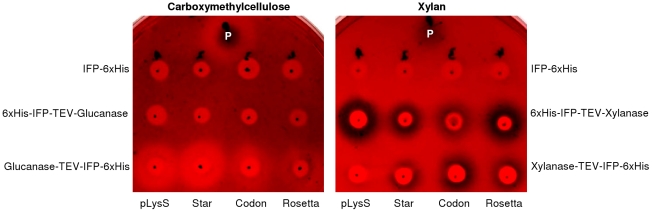
Enzymatic activity assays using glucanase- and xylanase-IFP fusion proteins expressed in *E. coli*. LIC-pDEST-LC1-/LC2 vectors encoding 6xHis-IFP-TEV-endo-β-1,4-glucanase/-xylanase and endo-β-1,4-glucanase-/xylanase-TEV-IFP-6xHis fusion proteins were transformed into the *E. coli* strains BL21 (DE3) pLysS (‘pLysS’), BL21 Star (DE3) pRARE (‘Star’), BL21 (DE3) CodonPlus-RIL (‘Codon’) and Rosetta (DE3) pRARE (‘Rosetta’). Enzymatic activity was tested by Congo Red staining and destaining with 1 M NaCl on carboxymethylcellulose- (left panel) or xylan- (right panel) containing agar plates after transferring 2 µL of the respective expression strains and over-night incubation at 37°C. Glucanase activity leads to the formation of a white halo around the colonies, whereas xylanase activity leads to the formation of a black halo [31]. *E. coli* cells expressing IFP-6xHis fusion protein were used as negative control, and cell-free supernatant of *Pichia pastoris* expression cultures containing secreted endo-β-1,4-glucanase-myc-6xHis or endo-β-1,4-xylanase-myc-6xHis fusion proteins were used as positive controls (P).

Two lignocellulolytic enzymes, endo-β-1,4-glucanase and endo-β-1,4-xylanase derived from the fungus *Emericella nidulans*, were used as test candidates for enzymatic activity assays. Both proteins were shown before to be active on carboxymethylcellulose (CMC) or xylan substrate after expression in *Pichia pastoris* as Myc-6xHis-tag fusion proteins [30]. To our knowledge both proteins were not expressed in *E. coli* before. Here we cloned the open reading frames of the two enzymes, via LIC, into the *E. coli* expression vectors LIC-pDEST-LC1 and -LC2. Resulting expression vectors encoding for a total of four IFP fusion proteins were transformed into different *E. coli* expression strains and investigated on CMC or xylan agar plates for enzymatic activity after Congo Red staining (**Fig. 12**). IFP-6xHis fusion protein-expressing *E. coli* strains were used as negative controls and *Pichia pastoris* strains expressing and secreting active endo-β-1,4-glucanase-myc-6xHis and endo-β-1,4-xylanase-myc-6xHis fusion proteins [30] were used as positive controls. On CMC agar plates (**Fig. 12**, left panel) characteristic clear zones were observed when endo-β-1,4-glucanase was expressed as endo-β-1,4-glucanase-TEV-IFP-6xHis fusion protein in all *E. coli* strains tested here (however, no glucanase activity was detected when the enzyme was fused to 6xHis-IFP-TEV at its N-terminus). On xylan-containing agar plates (**Fig. 12**, right panel) characteristic dark halos were observed when endo-β-1,4-xylanase was expressed in the different *E. coli* strains as fusions to IFP and 6xHis. Thus, it can be concluded that at least for the two enzymes tested here, fusions to IFP did not largely impair their enzymatic activity.

## Discussion

Efficient methods for the cloning, expression and functional analysis of proteins are highly wanted in functional genomics research. Here we established a LIC-IFP-based protein expression platform that combines various beneficial characteristics: very high (generally 100%) cloning efficiency due to a slightly modified LIC procedure (including a stuffer fragment between two LIC sites in the target vectors); multi-parallel, oriented insertion of LIC-enabled PCR fragments (obtained by only two separate PCR reactions) into different vectors for expression in prokaryotic and eukaryotic hosts and *in vitro*; simple detection of expressed proteins in intact cells by infrared imaging; facile infrared visualization of expressed proteins in crude protein extracts after denaturing SDS-PAGE directly in the cast gels. IFP not only serves as an exceptional marker for protein expression *in vivo* and *in vitro*, its excellent reporter properties may also trigger the development of new molecular and biochemical detection methods such as those reported here for the analysis of protein-protein and protein-DNA interactions.

In their original paper Shu *et al*. [20] reported successful expression of IFP in *E. coli*, human embryonic kidney cells (HEK293A), and mice. We recently demonstrated that IFP also functions as an excellent reporter for protein expression in the protozoan *Leishmania tarentolae* [21]. Here we show that IFP has similar beneficial properties in two further eukaryotic hosts, i.e. the yeasts *Kluyveromyces lactis* and *Pichia pastoris*. Although we have not tested other expression systems so far, we assume that IFP will out-perform as a novel reporter in other microbial systems as well. Furthermore, as shown in this report, functional IFP can be easily and rapidly reconstituted after *in vitro* transcription/translation.

Using the LIC-compatible vectors provided here, also untagged proteins can be produced. To this end, open reading frames are amplified with a reverse primer that includes a stop codon before cloning into LC2 vectors. Additionally, although not tested, the LIC-pIVEX-LC1/-LC2 vectors designed for *in vitro* expression in *E. coli*-derived transcription/translation extract may be modified to function in other T7-based *in vitro* expression systems, e.g. rabbit reticulocytes or wheat germ lysates. The LIC-IFP cloning and expression setup can also easily be extended to other vectors for expression in alternative hosts. In principal, LIC-IFP vectors could also be designed for protein expression in plants; however, plant cells often accumulate secondary metabolites and chlorophyll that intensely fluoresce when illuminated with infrared light used for IFP detection (not shown). Finally, the LIC-IFP vectors may be modified to include other cloning features, such as those realized in e.g. Golden Gate shuffling [6] or In-Fusion assembly (Clontech).

A further important result of our studies is that IFP classifies as an easy-to-handle reporter not only for the detection of protein expression in cells, but also for the visualisation of protein-protein and protein-DNA interactions *in vitro*. Protein-protein and protein-DNA interactions are key mechanisms for numerous biological functions in living cells and a suite of techniques has therefore been developed in the last decade to support the analysis of such interactions, including the yeast two- and one-hybrid systems, bimolecular fluorescence complementation, co-immunoprecipitation, DNA electrophoretic mobility shift assays, or pull-down assays, besides others. Here, we modified existing protein-protein and protein-DNA pull-down assays that traditionally detect interactions between bio-molecules by autoradiography using radioactively (^35^S-methionine) labeled proteins or by western blot using antibodies directed against the protein under analysis or an attached epitope tag. In our approach we appended IFP to proteins of interest and visualized their interactions with other proteins or DNA by infrared imaging in either cast protein gels (after SDS-PAGE) or in microtiter plates. Of note, with our IFP-based DNA-protein interaction assay we were able to demonstrate differential binding affinity to wild-type and mutated DNA *cis*-elements which closely matched our previous observations. We thus conclude that our novel protocol represents a simple and straightforward alternative for the confirmation of protein-protein and protein-DNA interactions that have e.g. been observed before in a yeast one-hybrid or CELD assay. Finally, using two cell wall-degrading enzymes as model proteins we demonstrated that our vector set allows rapid expression of IFP fusion proteins that retain catalytic activity.

## Supporting Information

Table S1 Primers used for the generation of LIC-IFP compatible expression vectors.
(DOC)

Table S2 Primers used for LIC of target open reading frames.
(DOC)

Table S3 Primers used for Gateway cloning of GRFs.
(DOC)
