# Supplementary material for: High-Throughput Protein Expression Using a Combination of Ligation-Independent Cloning (LIC) and Infrared Fluorescent Protein (IFP) Detection
Source: PLoS One. 2011 Apr 26;6(4):e18900. doi: 10.1371/journal.pone.0018900 (PMC3082538; doi:10.1371/journal.pone.0018900)
Supplement: Table S1 — Primers used for the generation of LIC-IFP compatible expression vectors. (DOC) [file pone.0018900.s001.doc]

# Supporting information

**Table S1.** Primers used for the generation of LIC-IFP compatible expression vectors.

| **Primer** | **Sequence of primer** |
| --- | --- |
| 269 | aagaagacatgtcgtactaccatcaccatcaccatcacgaatacgctg |
| 283 | agtaaacccgggtttatacagctcgtccattcc |
| 294 | ataagaatgcggccgcatggctcgggaccctctg |
| 295 | acccatgccgccgccctgaaaatacaggttctctttatacagctcgtccattcc |
| 296 | cagggcggcggcatgggttcttctgtttaaacctcagtgtcatcattcgaaag |
| 297 | ctggatccaggttctcgccctgtttaaacgctagcggattgaatggacg |
| 298 | taacccatgggttcttctgtttaaacctcagtgtcatcattcgaaag |
| 299 | gccgccctgaaaatacaggttctcgccctgtttaaacgctagcggattgaatggacg |
| 300 | acctgtattttcagggcggcggcggcatggctcgggaccctctg |
| 301a | atcaccatcaccatcacctcgaatcaatggctcgggaccctctg |
| 301b | accatggcacatatgtcgtactaccatcaccatcaccatcacctc |
| 302 | accatggcacatatgggttcttctgtttaaacctcagtgtcatcattcgaaag |
| 303 | gttggtacctttatacagctcgtccattcc |
| 304 | tagcccatgggctccggccatcaccatcaccatcacctc |
| 305 | ataagaatgcggccgcaggttctcgccctgtttaaacgctagcggattgaatggacg |
| 308 | ataagaataacgttcgaaacgatgggctccggccatcaccatcaccatcacctc |
| 309 | agtaaagtcgacaggttctcgccctgtttaaac |
| 310 | ataagaataacgttcgaaacgatgggttcttctgtttaaacc |
| 311 | agtaaagtcgactttatacagctcgtccattcc |
| 312 | aataacaagcttgaaaaaaatgggctccggccatcaccatcaccatcacctc |
| 313 | aataacaagcttgaaaaaaatgggttcttctgtttaaacc |
| 314a | tggtggtgatggtggtgggtacctttatacagctcgtccattcc |
| 314b | ataagaatgcggccgcctattagtggtggtgatggtggtgg |
