# Supplementary material for: High-Throughput Protein Expression Using a Combination of Ligation-Independent Cloning (LIC) and Infrared Fluorescent Protein (IFP) Detection
Source: PLoS One. 2011 Apr 26;6(4):e18900. doi: 10.1371/journal.pone.0018900 (PMC3082538; doi:10.1371/journal.pone.0018900)
Supplement: Table S2 — Primers used for LIC of target open reading frames. (DOC) [file pone.0018900.s002.doc]

# Supporting information

**Table S2.** Primers used for LIC of target open reading frames.

| **Gene** | **Gene code** | **Sequence of 5´-primer** | **Sequence of 3´-primer** |
| --- | --- | --- | --- |
| TPK1 | At5g55630 | tgggttcttctgtttcc[**a**tgtcgagtgatgcagctcgtac] | ggttctcgccctgttt**a**cc(ctatta)[cactcgcctgagattcg] |
| SAM1 | At1g02500 | tgggttcttctgtttcc[**a**tggagacttttctattcacatc] | ggttctcgccctgttt**a**cc(ctatta)[agcttgaggtttgtcccac] |
| ACO1 | At2g19590 | tgggttcttctgtttcc[**a**tggttttgatcaaagagag] | ggttctcgccctgttt**a**cc(ctatta)[ggctgaatccgcatttc] |
| ACS2 | At1g01480 | tgggttcttctgtttcc[**a**tgggtcttccgggaaaaaataaag] | ggttctcgccctgttt**a**cc(ctatta)[tgctcggagaagaggtgagtg] |
| ANAC042 | At2g43000 | tgggttcttctgtttcc[**a**tgagtggcgaaggtaacttagg] | ggttctcgccctgttt**a**cc(ctatta)[gggtttagtgttgccatctataac] |
| ANAC059 | At3g29035 | tgggttcttctgtttcc[**a**tggattacaaggtatcaagaagtg] | ggttctcgccctgttt**a**cc(ctatta)[gaatttccaaacgcaatcaagattc] |
| BGAL4 | At5g56870 | tgggttcttctgtttcc[**a**tctcttatgatcgtaaagctg] | ggttctcgccctgttt**a**cc(ctatta)[tccacggaacagaactctcg] |
| BGAL10 | At5g63810 | tgggttcttctgtttcc[**a**atcgggttactactgaaagc] | ggttctcgccctgttt**a**cc(ctatta)[gcctaatgtaaaattttgatgc] |
| Endo-β-1,4-glucanase | DQ490472 | tgggttcttctgtttcc[**a**tgaggtctctcgtccttct] | ggttctcgccctgttt**a**cc(ctatta)[ttgacttcccacgaaatacgg] |
| Endo-β-1,4-glucanase | DQ490490 | tgggttcttctgtttcc[**a**tggtctccttcaaatctctc] | ggttctcgccctgttt**a**cc(ctatta)[gtaaacagtaatagaagccgac] |

Gene-specific oligonucleotide sequences and double stop codons are labelled by squared and round parentheses. Underlined sequences correspond to LCA (5´-primer) and LCB (3´-primer) annealing sites. Adenine (**a**) indicates the position of thymine on the complementary strand required for the generation of LIC complementary overhangs.
