# Supplementary material for: High-Throughput Protein Expression Using a Combination of Ligation-Independent Cloning (LIC) and Infrared Fluorescent Protein (IFP) Detection
Source: PLoS One. 2011 Apr 26;6(4):e18900. doi: 10.1371/journal.pone.0018900 (PMC3082538; doi:10.1371/journal.pone.0018900)
Supplement: Table S3 — Primers used for Gateway cloning of GRFs. (DOC) [file pone.0018900.s003.doc]

# Supporting information

**Table S3.** Primers used for Gateway cloning of GRFs.

| **Gene** | **AGI code** | **Sequence of 5´-primer with *att*B1 sequence (**aaaaagcaggctta**) at the 5` end** | **Sequence of 3´-primer with *att*B2 sequence (**agaaagctgggta**) at the 3` end** |
| --- | --- | --- | --- |
| GRF1 | At4g09000 | atggcgacaccaggagcttc | ttaggattgttgctcgtcagc |
| GRF2 | At1g78300 | atggcgtctgggcgtgaag | ttactgctgttcctcggtcgg |
| GRF3 | At5g38480 | atgtcgacaagggaagagaatg | ttactcggcaccatcgggctttg |
| GRF4 | At1g35160 | atggcggcaccaccagcatc | ttagatctccttctgttcttcagc |
| GRF5 | At5g16050 | atgtcttctgattcgtccc | ttactgcgaaggtggtggttgg |
| GRF6 | At5g10450 | atggcggcgacattaggcag | ttaggcctcgtccatctgctcc |
